# Cobalt-mediated suppression of IFN-γ–JAK–STAT1 signaling reprograms IDO1-driven immunosuppression for metalloimmunotherapy

**DOI:** 10.1126/sciadv.aeg6398

**Published:** 2026-09-18

**Authors:** Tianyi Jiang, Xiaowan Huang, Zhixin Wu, Gulinaizaier Abudusaimaiti, Yang Song, Hongtao Duan, Yuping Zhou, Yaoxu Chen, Sitong Du, Hao Zhang, Jieying Qian, Yunjiao Zhang

**Affiliations:** ^1^ School of Medicine, South China University of Technology, Guangzhou, Guangdong 510006, China.; ^2^ Department of Diagnostic Radiology, Yong Loo Lin School of Medicine, National University of Singapore, Singapore 119074, Singapore.; ^3^ National Engineering Research Center for Tissue Restoration and Reconstruction and Key Laboratory of Biomedical Engineering of Guangdong Province, South China University of Technology, Guangzhou, Guangdong 511442, China.

## Abstract

Metal ions are increasingly recognized as regulators of immune function, yet their application in cancer immunotherapy remains underexplored. Here, we identified cobalt ions (Co^2+^) as potent suppressors of IFN-γ–induced IDO1 expression through systematic screening of biologically relevant metal ions. Across multiple cancer cell lines, Co^2+^ notably reduced IDO1 expression and kynurenine production. Mechanistically, Co^2+^ destabilized IFNGR1 and inhibited IFN-γ–JAK–STAT1 signaling, thereby restoring kynurenine/tryptophan metabolic balance and alleviating immunosuppression of CD8^+^ T cell. These effects reprogrammed the immunosuppressive tumor microenvironment toward enhanced cytotoxic T cell activity. To minimize the toxicity associated with free Co^2+^, we developed ConaHA, a hyaluronic acid–based nanoparticle platform enabling sustained and tumor-targeted cobalt delivery. ConaHA enhanced cobalt-mediated immune checkpoint blockade in vivo, resulting in notably improved antitumor efficacy in subcutaneous Panc02, MC38, and B16F10 tumor models and KPC (*LSL-Kras*^*G12D/+*^; *LSL-Trp53*^*R172H/+*^; *Pdx-1-Cre*) models. Collectively, these findings reveal a previously unrecognized immunoregulatory role of Co^2+^ and establish a promising framework for metalloimmunotherapy through modulation of metal-immune signaling pathways.

## INTRODUCTION

The immune system recognizes and eliminates external invaders, including tumor cells, within the tumor microenvironment (TME) under physiological conditions (*1*). However, tumor cells can evade immune surveillance by establishing multiple local immunosuppressive mechanisms, facilitating their survival at distinct stages of the antitumor immune response (*2*–*4*). By establishing local immunosuppressive networks, tumors attenuate distinct stages of the cancer-immunity cycle and progressively acquire resistance to cytotoxic lymphocyte-derived interferons (IFNs) (*5*–*6*). Among these cytokines, IFN-γ not only exerts potent immune-stimulatory effects but, like many cytokines, also activates intrinsic negative feedback mechanisms to limit excessive immune activation (*7*). Within tumor tissues, IFN-γ paradoxically promotes immunosuppression by inducing the expression of immunosuppressive mediators, including indoleamine 2,3-dioxygenase 1 (IDO1) and PD-L1 in tumor cells and tumor-associated macrophages, and promotes suppressor of cytokine signaling pathways in dendritic cells (DCs), collectively contributing to an immunosuppressive tumor milieu (*8*–*12*).

A principal conduit of this adaptive immune resistance is the kynurenine (KYN) pathway of tryptophan metabolism (*13*). Catalyzed by the heme-dependent dioxygenases IDO1, IDO2, and tryptophan 2,3-dioxygenase, this pathway governs the rate-limiting step of L-tryptophan oxidation (*14*–*15*). Among these enzymes, IDO1 has emerged as the dominant immunoregulatory node in cancer (*16*–*18*). By depleting tryptophan and generating bioactive KYN metabolites, IDO1 imposes metabolic constraints on T cell proliferation and effector function, thereby licensing tumor immune escape (*19*–*21*). Elevated IDO1 expression correlates with adverse clinical outcomes across diverse malignancies, underscoring its appeal as a therapeutic target (*22*). However, direct pharmacological inhibition of IDO1 has yielded limited clinical benefit, highlighting the need for alternative strategies to modulate this axis.

Metal ions constitute a pervasive yet underappreciated layer of immune regulation, and metal-based therapeutics have a long-standing track record of clinical success in treating diverse diseases (*23*, *24*). As cofactors for nearly half of all enzymes, metals participate in virtually every core biological process (BP), with transition metals such as iron, zinc, manganese, copper, and cobalt leveraging redox activity and coordination chemistry to shape signaling and metabolism (*25*–*27*). Their immunological roles span pathogen defense (*28*, *29*), lymphocyte activation (*30*, *31*), stem cell maintenance (K^+^) (*32*, *33*), inflammasome assembly (*34*, *35*), and innate immune sensing pathways, including cGAS-STING signaling (*36*–*38*). These insights have catalyzed the emergence of metalloimmunotherapy, an approach that seeks to harness the regulatory capacity of metals to recalibrate immune responses in disease.

Cobalt is an essential micronutrient required for vitamin B_12_ biosynthesis and has established functions in innate immunity, yet its impact on adaptive immune regulation remains poorly defined. Given the centrality of the IFN-γ–IDO1 axis in tumor immune evasion and the chemical versatility of cobalt, we systematically interrogated the immunomodulatory potential of biologically relevant metal ions. We identify divalent cobalt (Co^2+^) as a potent suppressor of IFN-γ–induced IDO1 expression in tumor cells, which directly binds to IFNGR1, the main receptor of IFN-γ, induces its degradation, and disables downstream IFN-γ–Janus kinase (JAK)–signal transducer and activator of transcription 1 (STAT1) signaling. By restoring KYN-tryptophan metabolic balance, Co^2+^ alleviates tumor-associated immunosuppression and reinvigorates CD8^+^ T cell [cytotoxic T lymphocyte (CTL)] and DC function. To mitigate the systemic toxicity associated with free cobalt, we engineered a hyaluronic acid (HA)–based nanoparticle platform enabling sustained and localized Co^2+^ delivery, thereby enhancing the therapeutic index of cobalt-mediated immune checkpoint modulation in multiple tumor-bearing mouse models. Collectively, our work establishes a framework for cobalt-directed metalloimmunotherapy and expands the conceptual repertoire for targeting metabolic immune checkpoints in cancer.

## RESULTS

### Co^2+^ suppresses IFN-γ–induced IDO1 activity and reverses tumor-associated immunosuppression in vitro

**Fig. 1.**
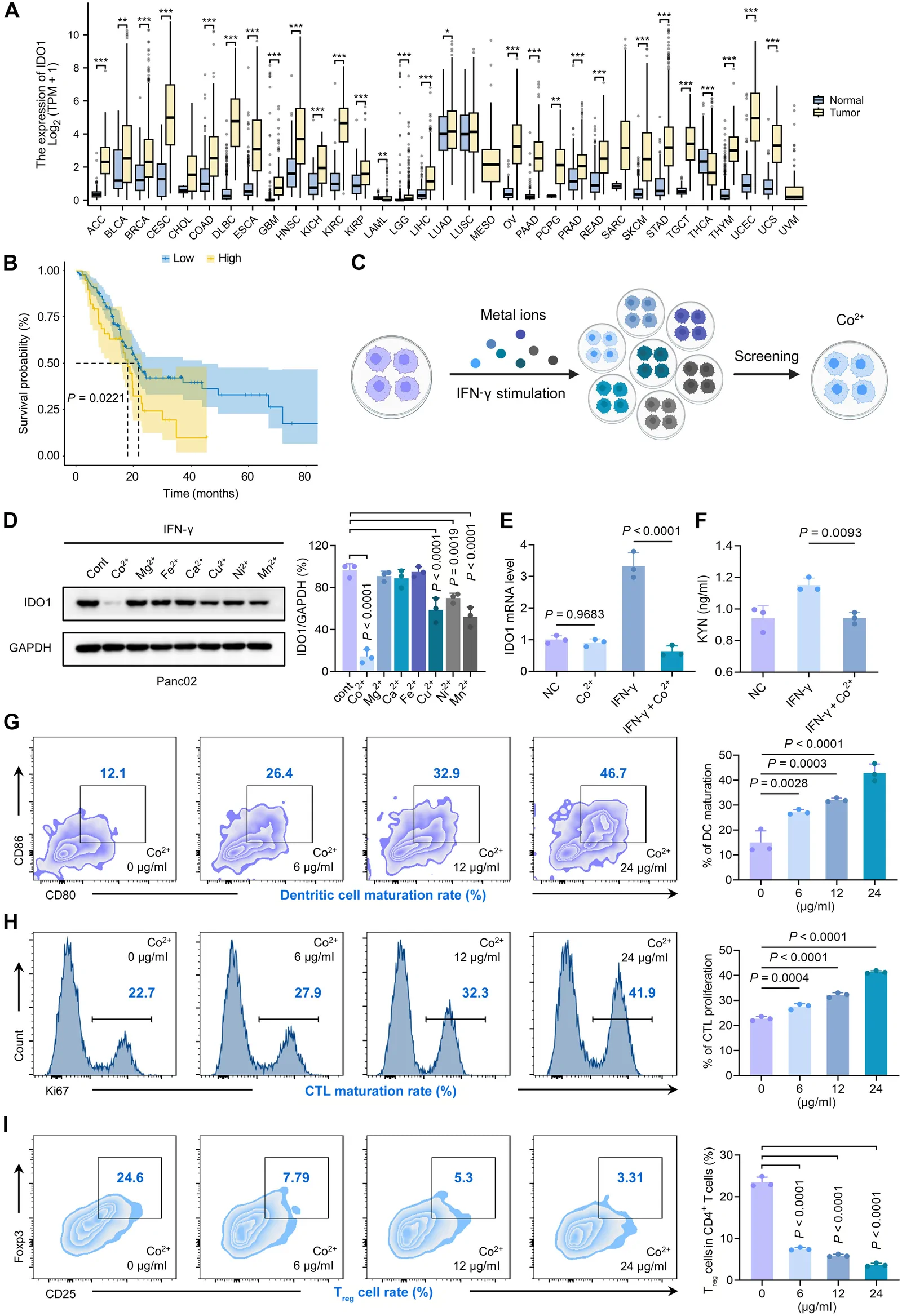
Co^2+^ inhibits IFN-γ–induced IDO1 activity and immunosuppression in vitro. (**A**) Comparison of IDO1 expression levels between different cancer types and normal tissues. (**B**) Kaplan-Meier curves for survival probability of patients with pancreatic cancer with low versus high expression of IDO1. (**C**) Screening for inhibition of IDO1 expression by different metal ions. (**D**) Western blot and quantitative analysis of IDO1 levels in Panc02 cells treated with different metal ions (Co^2+^, Mg^2+^, Fe^2+^, Ca^2+^, Cu^2+^, Ni^2+^, and Mn^2+^) at 200 μM under IFN-γ stimulation (50 ng/ml). (**E**) Quantitative RT-PCR analysis of IDO1 mRNA level in Panc02 cells treated with or without Co^2+^ under IFN-γ stimulation. Phosphate-buffered saline (PBS)–treated cells without IFN-γ stimulation served as the negative control (NC). (**F**) Enzyme-linked immunosorbent assay (ELISA) analysis of KYN levels in supernatants of Panc02 cells treated with or without Co^2+^ under IFN-γ stimulation. (**G** to **I**) Flow cytometry and quantitative analysis of CD80^+^CD86^+^ DCs, Ki67^+^ CTLs, and CD25^+^Foxp3^+^ T_reg_ cells after incubation with Co^2+^-treated Panc02 cells under IFN-γ stimulation for 24 hours (G) or 72 hours (H and I). Data are presented as mean ± SD (*n* = 3).

To evaluate the clinical relevance of IDO1, we interrogated transcriptomic datasets from The Cancer Genome Atlas (TCGA). Bioinformatic analysis revealed that IDO1 expression was significantly up-regulated across 28 tumor types, including pancreatic adenocarcinoma (PAAD), relative to matched normal tissues (Fig. 1A). Notably, survival analysis stratified by IDO1 expression levels showed that patients with PAAD and low IDO1 expression were associated with improved overall survival (Fig. 1B), supporting its functional importance in tumor progression and underscoring the need for strategies that modulate IDO1 expression. To identify pharmacologically tractable regulators of IDO1, we performed a focused screen of biologically relevant metal ions, assessing their capacity to suppress IFN-γ–induced IDO1 protein expression. Among the candidates tested, Co^2+^, Cu^2+^, Ni^2+^, and Mn^2+^ markedly reduced IDO1 abundance in IFN-γ–stimulated Panc02 cells (Fig. 1, C and D). Co^2+^ exhibited the most pronounced inhibitory effect and was therefore selected for further investigation.

Co^2+^ consistently suppressed IFN-γ–induced IDO1 expression across multiple murine cancer cell lines, including Panc02, MC38, B16F10, and KPC (fig. S1, A to E), indicating a broad and tumor-agnostic activity. At the transcriptional level, Co^2+^ did not alter basal IDO1 mRNA expression in the absence of IFN-γ but significantly attenuated IFN-γ–driven IDO1 induction, as determined by quantitative reverse transcription polymerase chain reaction (RT-PCR) (Fig. 1E). This context-dependent effect suggests that Co^2+^ selectively interferes with IFN-γ–responsive transcriptional programs. Consistent with these findings, enzyme-linked immunosorbent assays (ELISAs) revealed a marked reduction in KYN accumulation in culture supernatants following Co^2+^ treatment (Fig. 1F), demonstrating that suppression of IDO1 expression translated into diminished enzymatic activity.

Given the central role of IDO1 in shaping immune tolerance through tryptophan depletion and KYN production, we next assessed whether Co^2+^ could restore immune competence in vitro. Using a coculture system comprising Panc02 tumor cells and primary murine immune cells, we evaluated DC maturation, regulatory T cell (T_reg_ cell) differentiation and CTL proliferation (*39*, *40*). Co^2+^ treatment dose-dependently enhanced DC maturation and CTL expansion, as reflected by increased frequencies of CD86^+^CD80^+^ DCs and Ki67^+^ CTLs (Fig. 1, G and H). At the highest concentration tested, Co^2+^ increased mature DCs to 46.7% (3.86-fold over control) and proliferating CTLs to 41.9% (1.85-fold over control). In parallel, the proportion of T_reg_ cells was markedly reduced, declining to 0.13-fold relative to control (Fig. 1I). Collectively, these findings establish Co^2+^ as a potent inhibitor of IFN-γ–induced IDO1 expression and activity. By constraining KYN pathway signaling, Co^2+^ reprograms the tumor-immune interface, promoting DC activation, limiting T_reg_ cell differentiation, and enhancing cytotoxic T cell expansion. These coordinated immunomodulatory effects provide mechanistic evidence that cobalt counteracts metabolic immune suppression and potentiates antitumor immunity in vitro.

### Co^2+^ alleviates IDO1/PD-L1–driven immune suppression of CD8^+^ T cells

**Fig. 2.**
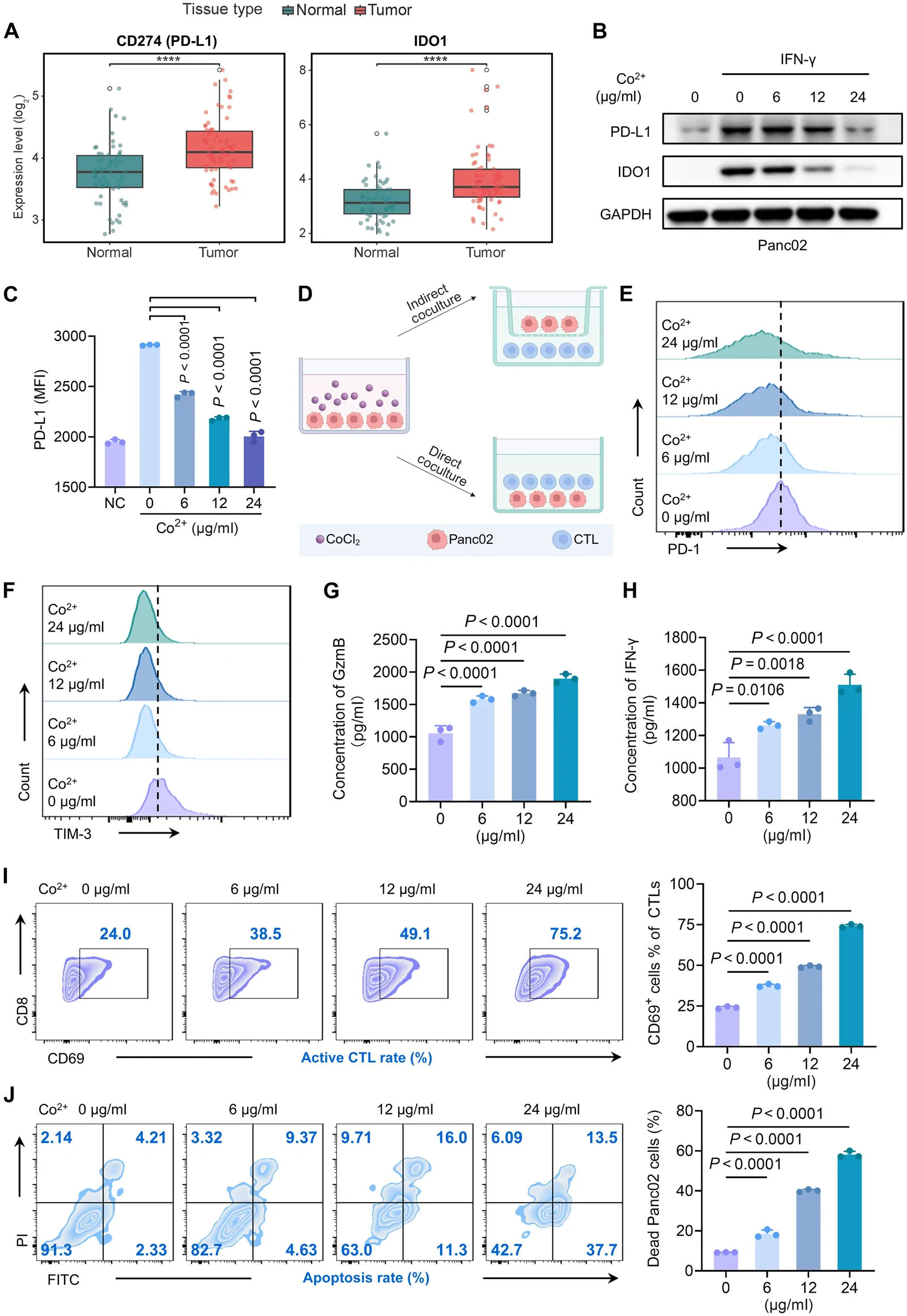
Co^2+^ alleviates IDO1/PD-L1–driven immune suppression of CD8^+^ T cells. (**A**) CD274 (PD-L1) and IDO1 expression in pancreatic cancer versus normal pancreatic via the GEO database (GSE62452). (**B**) Western blot analysis of PD-L1 and IDO1 levels in Panc02 cells treated with Co^2+^ (0, 6, 12, and 24 μg/ml) under IFN-γ stimulation. GAPDH, glyceraldehyde phosphate dehydrogenase. (**C**) Flow cytometry analysis of PD-L1 levels in Panc02 cells treated with Co^2+^ (0, 6, 12, and 24 μg/ml) under IFN-γ stimulation. PBS-treated cells without IFN-γ stimulation served as the negative control (NC). (**D**) Indirect and direct coculture of CTLs with Panc02 cells preincubated with Co^2+^ under IFN-γ stimulation for 24 hours. (**E** and **F**) Flow cytometry analysis of PD-1^+^ CTLs and TIM-3^+^CTLs after indirect coculture with IFN-γ– and Co^2+^-treated Panc02 cells for 72 hours. (**G** and **H**) ELISA analysis of the GzmB (G) and IFN-γ (H) levels in coculture supernatants. (**I**) Flow cytometry and quantitative analysis of CD69^+^CTLs after indirect coculture with IFN-γ– and Co^2+^-treated Panc02 cells for 72 hours. (**J**) Cell death analysis of IFN-γ– and Co^2+^-treated Panc02 cells direct coculture with activated CTLs separated from C57BL/6J mouse spleen by flow cytometry. Data are presented as mean ± SD (*n* = 3).

Given the central role of IFN-γ signaling in coordinating immunosuppressive circuits, we interrogated Gene Expression Omnibus (GEO) datasets and found that both IDO1 and PD-L1 were significantly up-regulated in pancreatic ductal adenocarcinoma (PDAC) relative to normal pancreatic tissue (Fig. 2A). Furthermore, correlation analysis confirmed a significant positive correlation between PD-L1 and IDO1 expression, with a Pearson correlation coefficient of 0.570 (fig. S2A). These data suggest the convergence of multiple IFN-γ–responsive immune checkpoints within the TME. Having established that Co^2+^ suppresses IFN-γ–induced IDO1 expression, we next examined its impact on PD-L1. In IFN-γ–stimulated Panc02 cells, Co^2+^ markedly and dose-dependently reduced PD-L1 protein abundance (Fig. 2, B and C, and fig. S2, B and C), indicating that cobalt broadly constrains IFN-γ–driven adaptive immune resistance programs.

Since IDO1 and PD-L1 cooperatively promote CD8^+^ T cell exhaustion, which is a dysfunctional state marked by sustained inhibitory receptor expression and impaired effector function, we next examined whether Co^2+^ treatment could restore CD8^+^ T cell function. Using both direct and indirect coculture systems comprising Co^2+^-treated Panc02 cells and primary CD8^+^ T cells (Fig. 2D), we evaluated phenotypic and functional hallmarks of exhaustion. Co^2+^ treatment significantly reduced the proportion of exhausted CD8^+^ T cells, as reflected by decreased expression of PD-1 and TIM-3 (Fig. 2, E and F, and fig. S2D). Functional recovery was corroborated by enhanced effector cytokine production. ELISA assays revealed dose-dependent increases in granzyme B (GzmB) and IFN-γ secretion by CD8^+^ T cells exposed to Co^2+^-treated tumor cells (Fig. 2, G and H), consistent with restoration of cytotoxic capacity. In parallel, expression of the early activation marker CD69 was significantly up-regulated (Fig. 2I), indicating a phenotypic transition of CD8^+^ T cells from an exhausted to an activated state. Moreover, the exogenous KYN supplementation substantially abolished the potent antitumor immune effect mediated by Co^2+^ intervention, indicating that the antitumor immunomodulatory effect of Co^2+^ is tightly dependent on its suppression of the IDO1-KYN pathway (fig. S2E). Last, CD8^+^ T cell–mediated cytotoxicity assays demonstrated that Co^2+^ treatment significantly enhanced the killing capacity of CD8^+^ T cells against Panc02 cells, with cytolytic activity increasing from 8.7% in the control group to 57.3% at the highest Co^2+^ concentration tested (Fig. 2J).

Together, these findings demonstrate that Co^2+^ simultaneously suppresses IFN-γ–driven IDO1 and PD-L1 expression and alleviates CD8^+^ T cell exhaustion. By dismantling coordinated metabolic and checkpoint-mediated inhibitory pathways, cobalt restores effective cytotoxic T cell responses and reinforces antitumor immunity in vitro.

### Co^2+^ inhibits JAK/STAT signaling and IFN-γ–induced STAT1 activation by directly targeting IFNGR1

**Fig. 3.**
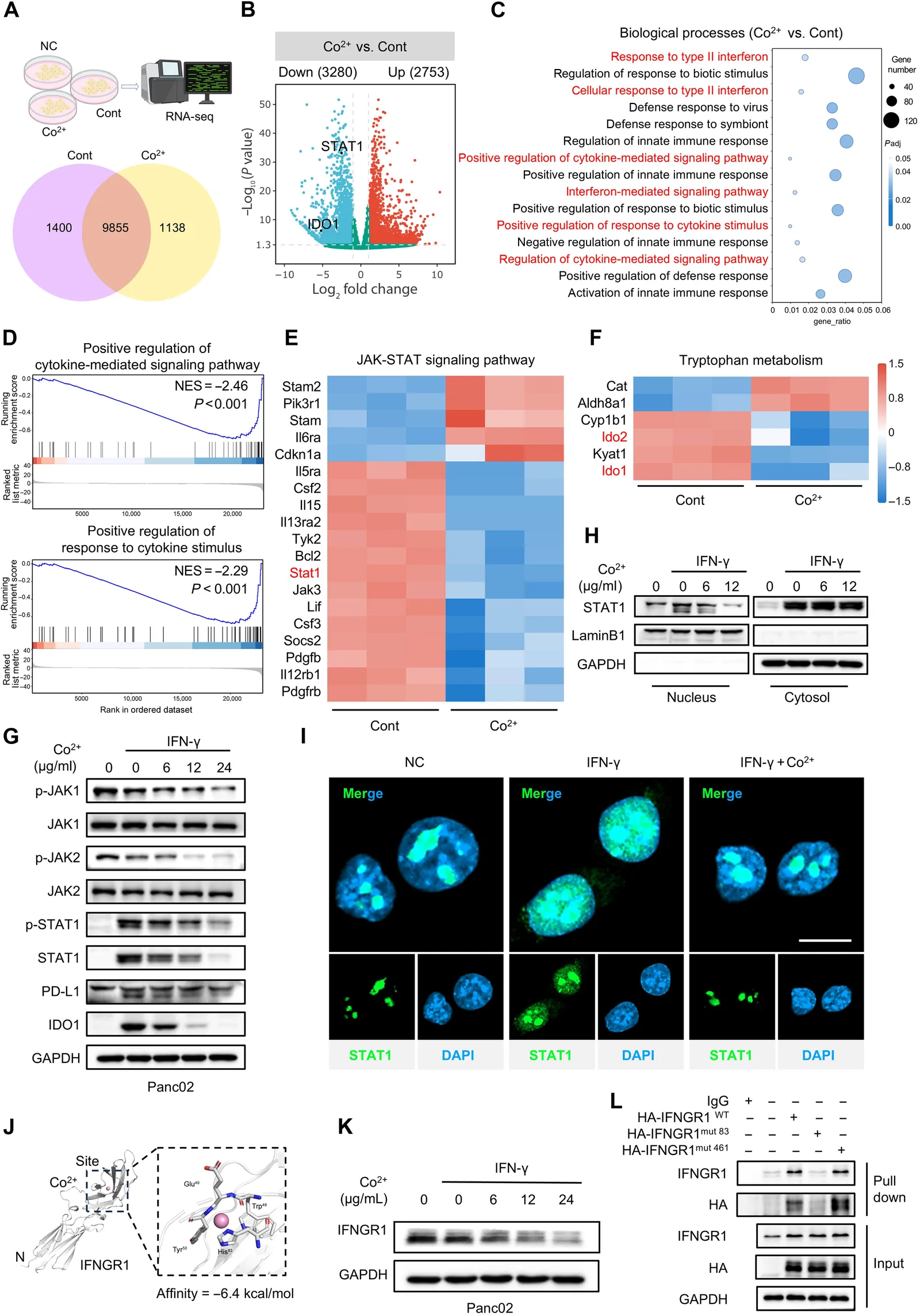
Co^2+^ Inhibits JAK/STAT signaling and IFN-γ–induced STAT1 activation by directly targeting IFNGR1. (**A**) RNA sequencing (RNA-seq) analysis of Panc02 cells treated with PBS (Cont) or Co^2+^ (24 μg/ml) under IFN-γ stimulation (*n* = 3). PBS-treated cells without IFN-γ stimulation served as the negative control (NC). (**B**) Volcano plot showing DEGs between Co^2+^ and Cont. (**C**) Top GO terms of BP enriched among down-regulated DEGs (Co^2+^ versus Cont) from RNA-seq data. The top 15 terms were screened and presented according to the smaller *P* value. (**D**) GSEA plots associated with cytokine signaling pathway (Co^2+^ versus Cont). (**E** and **F**) Heatmap showing DEGs in the JAK/STAT signaling pathway and the tryptophan metabolism signaling pathway (*P*adj < 0.001, |log_2_ fold change| > 1.5). (**G**) Western blot analyses of the indicated proteins in Panc02 cells treated with Co^2+^ (0, 6, 12, and 24 μg/ml) upon IFN-γ stimulation. (**H**) Western blot analysis of STAT1 from nuclear fraction in Panc02 cells treated with Co^2+^ (0, 6, and 12 μg/ml) with IFN-γ stimulation. (**I**) Representative immunofluorescence images staining for STAT1 in IFN-γ– and IFN-γ + Co^2+^–treated Panc02 cells with IFN-γ stimulation. The Alexa Fluor 488 was used to label STAT1, and the nucleus was stained with 4′,6-diamidino-2-phenylindole (DAPI). Scale bars, 20 μm. (**J**) Molecular docking model of IFNGR1 with Co^2+^. (**K**) Western blot analysis of IFNGR1 protein level in Panc02 cells treated with Co^2+^ (0, 6, 12, and 24 μg/ml) upon IFN-γ stimulation. (**L**) Pull-down assays to compare the binding of Co^2+^ to IFNGR1 wild-type and mutants. HEK-293T cells were transfected with plasmids encoding HA-IFNGR1^WT^, HA-IFNGR1^H83A^, or HA-IFNGR1^H461A^. IFNGR1 proteins were pulled down using Co NTA beads. Western blots of input and eluted proteins were probed with an anti-IFNGR1 antibody. HA-IFNGR1^H461A^ as a negative control. Data are presented as mean ± SD (*n* = 3). IgG, immunoglobulin G.

To identify the molecular pathways underlying Co^2+^-mediated suppression of IFN-γ–induced immunosuppressive programs, we performed global transcriptional profiling analysis of Panc02 cells under IFN-γ stimulation in the presence or absence of Co^2+^ (Fig. 3A). Differential expression analysis revealed that both IDO1 and STAT1, a key regulator orchestrating IFN-γ–driven IDO1 transcription, were significantly down-regulated upon Co^2+^ treatment, as illustrated by the volcano plot (Fig. 3B). To delineate the BPs induced by Co^2+^ treatment, Gene Ontology (GO) enrichment analysis was conducted on down-regulated differentially expressed genes (DEGs; *P* < 0.05, fold change > 2) identified from the comparison between the Co^2+^ group and the Cont group. Notably, multiple BP terms associated with type II IFN signaling and cytokine responses pathways were significantly enriched (Fig. 3, C and D), indicating that Co^2+^ broadly attenuates IFN-γ–responsive transcriptional networks. Consistent with this observation, pathway-level interrogation revealed that genes within both the JAK-STAT pathway and the tryptophan metabolism pathway were predominantly down-regulated following Co^2+^ treatment (Fig. 3, E and F). These data implicate inhibition of canonical IFN-γ signaling as a principal mechanism by which Co^2+^ restrains IDO1 expression. IFN-γ binding to its cognate receptor (IFNGR) triggers activation of receptor-associated JAK1 and JAK2, culminating in phosphorylation of STAT1, its nuclear translocation, and transcription of IFN-stimulated genes. Western blot analysis confirmed that Co^2+^ reduced phosphorylation of JAK-STAT pathway components of Panc02 and KPC cells (Fig. 3G and fig. S3, A and B). Furthermore, nuclear fractionation and confocal immunofluorescence demonstrated a marked decrease in STAT1 nuclear accumulation in Co^2+^-treated cells (Fig. 3, H and I), consistent with impaired transcriptional activation. To further delineate the proximal molecular mechanism underlying JAK-STAT pathway inhibition, we next investigate whether Co^2+^ directly targets components of the IFN-γ receptor complex. Structural modeling using AlphaFold2 to predict the three-dimensional architecture of IFNGR1 protein, followed by molecular docking analysis, identified His-83 as a putative extracellular Co^2+^ coordination site (Fig. 3J). Notably, quantitative RT-PCR analysis demonstrated that Co^2+^ did not alter IFNGR1 mRNA levels (fig. S3D), whereas Western blot revealed a marked reduction in IFNGR1 protein levels under IFN-γ stimulation in the presence of Co^2+^ (Fig. 3K and fig. S3, E and F). Further in vitro pull-down assays were performed to verify the cobalt-binding sites of IFNGR1. Compared with wild-type IFNGR1 (IFNGR1^WT^), the cobalt-binding ability of IFNGR1^H83A^ was distinctly diminished (Fig. 3L), demonstrating that cobalt predominantly interacts with IFNGR1 via the His-83 residue. Cycloheximide chase assays confirmed that Co^2+^ modulates IFNGR1 expression at the posttranscriptional level, specifically by facilitating its protein degradation (fig. S3G). The lysosome inhibitor chloroquine, rather than the proteasome inhibitor MG132, effectively abolished Co^2+^-induced IFNGR1 degradation (fig. S3, H and I), demonstrating that this degradation process relies exclusively on the lysosome pathway. However, equivalent Co^2+^ treatment failed to alter IFNGR1 abundance in lymphocytes, RAW264.7 macrophages, bone marrow–derived macrophages (BMDMs), and bone marrow–derived dendritic cells (BMDCs) (fig. S3J). These findings suggest that Co^2+^ preferentially suppresses IFNGR1 signaling in tumor cells with negligible impacts on immune cells, likely owing to differential IFNGR1 surface expression.

Collectively, these data support that Co^2+^ binds to the extracellular His-83 residue of IFNGR1 and promotes its degradation via the lysosome pathway. By targeting the upstream IFNGR1-JAK-STAT signaling axis, Co^2+^ markedly suppresses IFN-γ–triggered STAT1 phosphorylation and nuclear translocation. This signaling blockade further restricts the transcription of IDO1 and PD-L1, thereby restraining the KYN immunosuppressive pathway and reversing tumor cell–intrinsic adaptive immune resistance.

### Design and characterization of ConaHA for tumor-targeted cobalt delivery

**Fig. 4.**
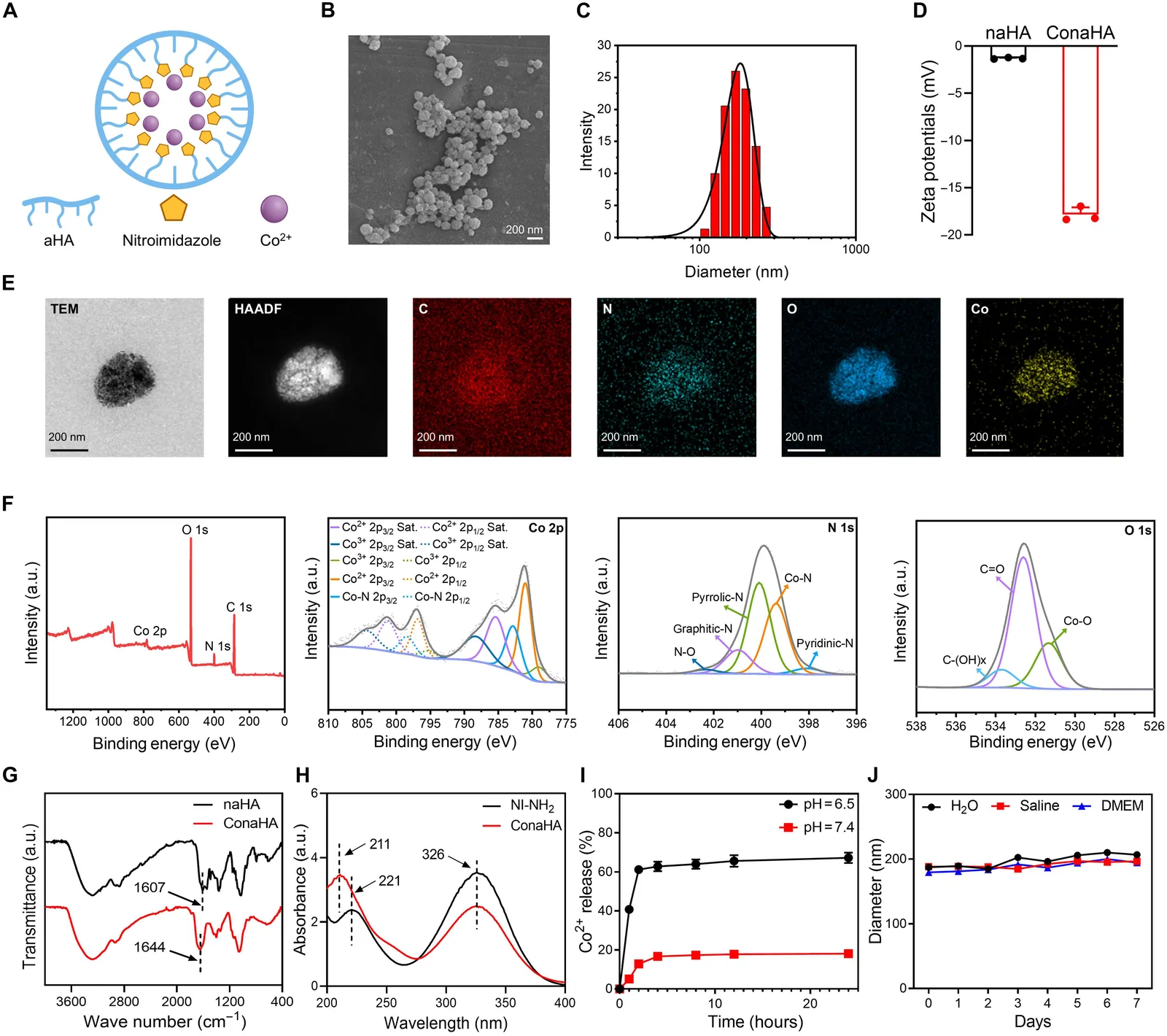
Preparation and characterization of ConaHA for targeted delivery of Co ions. (**A**) Schematic illustration of the construction of ConaHA. (**B**) Scanning electron microscopy image of ConaHA. (**C**) Size distribution of ConaHA in water as measured by dynamic light scattering. (**D**) Zeta potentials of naHA and ConaHA. (**E**) Energy-dispersive spectroscopy (EDS) elemental mapping distribution of ConaHA of the surface elemental percentage of C, N, O, and Co in ConaHA. (**F**) The total XPS survey spectrum and individual elemental high-resolution Co 2p, N 1s, and O 1s spectra of ConaHA. (**G**) Fourier transform infrared spectroscopy of naHA and ConaHA. (**H**) UV-vis absorption spectrum analysis of NI-NH_2_ and ConaHA. (**I**) Co^2+^ release curve from ConaHA in PBS at pH 7.4 and pH 6.5. (**J**) Hydrodynamic diameter distribution of ConaHA measured over 7 days in different solutions. Data are presented as mean ± SD (*n* = 3). HAADF, high-angle annular dark field.

Given the systemic toxicity associated with free Co^2+^ exposure, we sought to develop a tumor-targeted delivery strategy capable of confining cobalt activity to the TME. Thus, we engineered an acid-responsive, HA-based nanoplatform (ConaHA) for controlled Co^2+^ release. The synthetic route is outlined in Fig. 4A. Briefly, HA was first oxidized to generate aldehyde-modified HA (aHA), followed by conjugation with hypoxia-responsive 2-nitroimidazole (2-NI) to yield NI-grafted aHA (naHA). Coordination-driven self-assembly of naHA with Co^2+^ afforded ConaHA nanoparticles, designed to exploit both hypoxic responsiveness and HA-mediated tumor targeting (fig. S4A).

Morphological and physicochemical analyses confirmed successful nanoparticle formation. Scanning electron microscopy and dynamic light scattering revealed that ConaHA exhibited a uniform spherical morphology with an average hydrodynamic diameter of ∼200 nm (Fig. 4, B and C), a size range compatible with enhanced permeability and retention in tumors. Zeta potential measurements further indicated a negatively charged surface, with an average potential of −16.96 mV (Fig. 4D). High-angle annular dark-field scanning transmission electron microscopy, coupled with energy-dispersive spectroscopy elemental mapping demonstrated uniform spatial distribution of carbon (C), nitrogen (N), oxygen (O), and cobalt (Co) throughout the nanoparticles, confirming uniform cobalt incorporation (Fig. 4E). The x-ray photoelectron spectroscopy (XPS) spectrum further validated successful coordination. The Co 2p spectrum exhibited characteristic Co^2+^ peaks at 781.02 and 796.86 eV with corresponding satellite features, while N 1s and O 1s spectra supported Co^2+^ coordination, indicating successful complex formation (Fig. 4F). Fourier transform infrared spectroscopy revealed a shift of the ─C═N stretching vibration from 1607 cm^−1^ in naHA to 1644 cm^−1^ in ConaHA, indicative of Co^2+^ coordination (Fig. 4G). The ─CHO stretching vibrations peak appeared at 1730 cm^−1^ in aHA compared to HA, proving that aHA provided more reaction sites for NI-NH_2_ than HA (fig. S4B). Ultraviolet-visible spectroscopy showed blue-shifted absorption peaks at 211 and 326 nm compared with free 2-NI-NH_2_, consistent with altered electronic transitions arising from supramolecular assembly (Fig. 4H).

Inductively coupled plasma atomic emission spectroscopy demonstrated pH-responsive cobalt release. At mildly acidic pH (6.5), mimicking the TME, 67.17% of Co^2+^ was released within 24 hours, whereas only 18.07% was released under physiological conditions (pH 7.4) (Fig. 4I), indicating tumor-selective release behavior. In addition, ConaHA exhibited excellent colloidal stability in physiologically relevant media, with negligible changes in hydrodynamic size over time (Fig. 4J), indicating its suitability for biological applications. Functionally, ConaHA suppressed IDO1 expression to an extent comparable to free Co^2+^ at equivalent cobalt concentrations (fig. S4C), demonstrating preserved bioactivity. Notably, pretreatment with the HA-specific CD44 inhibitor iCD44 completely eliminated the ConaHA-mediated IDO1 down-regulation (fig. S4D), confirming that the biological activity of ConaHA is dependent on HA-facilitated cellular uptake. Collectively, these results demonstrate the successful construction, structural integrity, and tumor-responsive release properties of ConaHA, providing a rational platform for targeted cobalt delivery and subsequent in vitro and in vivo evaluation.

### ConaHA exerts robust antitumor activity in vitro

To determine whether the antitumor effects of ConaHA extend beyond modulation of the IFN-γ–STAT1 signaling axis, we systematically assessed its direct impact on tumor cell viability and malignant phenotypes. Cytocompatibility assays demonstrated negligible toxicity towards nonmalignant cells, including L929 murine fibroblasts and BMDMs, indicating favorable biocompatibility (fig. S5A). In contrast, ConaHA induced marked cytotoxicity across multiple tumor cell lines, including Panc02, MC38, and B16F10. Following 24-hour exposure at a cobalt-equivalent concentration of 20 μg/ml, tumor cell viability declined to below 50% of untreated controls, underscoring broad-spectrum antitumor activity. We next examined clonogenic potential and migratory capacity to assess effects on tumor cell proliferation and invasiveness. At equivalent cobalt concentrations, ConaHA suppressed colony formation and cell migration more effectively than free Co^2+^(fig. S5, B to D), indicating that nanoparticle-mediated delivery enhances antitumor potency. The cell apoptosis rate of ConaHA-treated cells was assessed in Panc02 and B16F10 cells via annexin V–fluorescein isothiocyanate (FITC)/propidium iodide staining. As shown in fig. S5E, naHA induced minimal apoptosis compared with the untreated group, indicating negligible intrinsic cytotoxicity. By contrast, ConaHA significantly increased apoptotic cell death, consistent with efficient intracellular delivery and localized cobalt release. Notably, the proapoptotic effect of ConaHA exceeded that of free Co^2+^ at matched concentrations, highlighting the functional advantage conferred by the nanocarrier system. Together, these findings demonstrate that ConaHA exerts robust, tumor-selective cytotoxic effects in vitro, suppressing proliferation and migration while promoting apoptosis. These antitumor activities occur independently of IFN-γ–STAT1 signaling modulation and provide a mechanistic foundation for subsequent in vivo evaluation of therapeutic efficacy and safety.

### Antitumor efficacy of ConaHA in Panc02 tumor–bearing mice

**Fig. 5.**
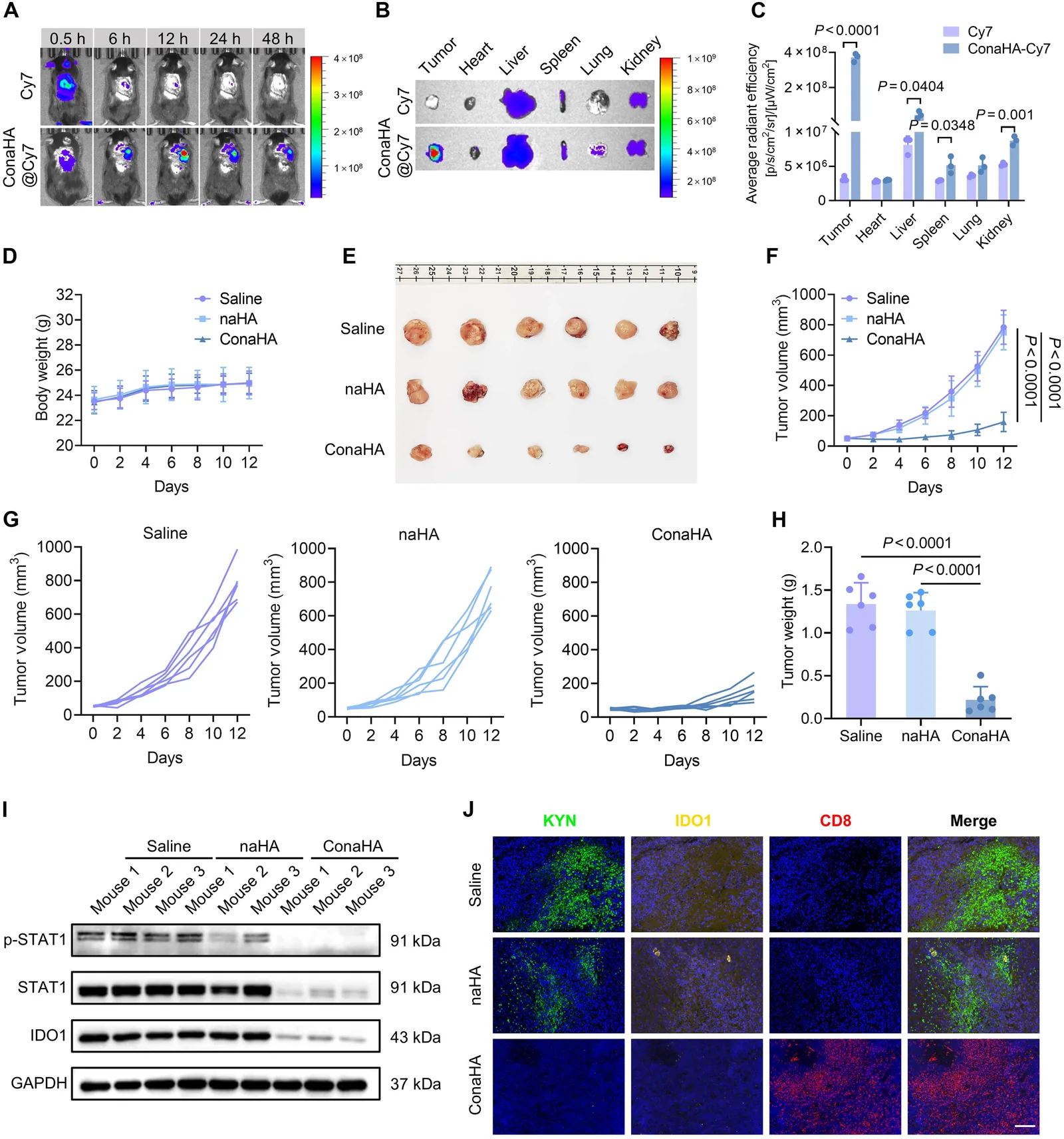
Antitumor efficacy of ConaHA in Panc02 tumor–bearing mice. (**A**) In vivo fluorescence imaging of Panc02 tumor–bearing mice at 0.5, 6, 12, 24, and 48 hours after tail vein injection of ConaHA. (**B** and **C**) Ex vivo representative fluorescence distribution and quantitative analysis of major organs collected at 48 hours using in vivo imaging system (*n* = 3). (**D**) Body weight of the mice in the various groups (*n* = 6 per group). (**E**) Appearance of tumors in different groups of mice. (**F** and **G**) Mean and individual tumor growth curves in mice subjected to different treatments (*n* = 6). (**H**) Average tumor weight of various groups (*n* = 6). (**I**) Western blot of p-STAT1, STAT1, and IDO1 levels in tumor tissues of the mice in the various groups. (**J**) Representative immunofluorescence images in tumor tissues of the mice stained with anti-KYN (labeled with Alexa Fluor 488), anti-IDO1 (labeled with Cy5), and anti-CD8 (labeled with Cy3) antibodies in the various groups. Scale bar, 100 μm. Data are presented as mean ± SD.

Building on the capacity of Co^2+^ to modulate KYN/TRP metabolism and alleviate immunosuppression in vitro, we systematically evaluated the therapeutic efficacy of ConaHA in a poorly immunogenic Panc02 tumor–bearing mouse model. Tumor-targeting capability was first verified by in vivo fluorescence imaging. Following systemic administration, ConaHA exhibited sustained tumor accumulation, with robust Cy7 signals persisting for up to 48 hours (Fig. 5, A to C), consistent with efficient tumor homing. Next, we further detected cobalt distribution in major organs following treatment with free Co^2+^ and ConaHA. Higher cobalt accumulation was observed in the free Co^2+^ group compared with the ConaHA group (fig. S6A). Comprehensive biosafety assessment demonstrated favorable tolerability. Even at a concentration of 160 μg/ml, ConaHA induced only 0.7% hemolysis of red blood cells, supporting good hemocompatibility (fig. S6B). Further in vivo experiments verified that only free Co^2+^ caused obvious body weight loss in mice, whereas no significant changes in body weight were observed in the ConaHA group (fig. S6C), confirming that ConaHA exhibits good biocompatibility. In addition, hematological and serum biochemical analyses revealed that no significant differences were observed in alanine aminotransferase, aspartate transaminase, blood urea nitrogen, or serum creatinine levels compared with normal mice. Hematoxylin and eosin staining of major organs (including heart, liver, spleen, lung, and kidney) also showed no pathological abnormalities, confirming good systemic compatibility of ConaHA (fig. S6, D and E).

To assess a therapeutic benefit, Panc02-bearing C57BL/6J mice were randomized to receive saline, naHA, or ConaHA (*n* = 6 per group). Nanoparticles were administered intravenously every 2 days at an equivalent HA dose of 40 mg/kg. Body weight remained stable across groups (Fig. 5D), further supporting tolerability at the tested dose. Notably, ConaHA suppressed tumor growth compared with both saline and naHA controls, whereas naHA alone exerted no measurable antitumor effect (Fig. 5, E to H). Mechanistically, we examined whether ConaHA restored metabolic and immune homeostasis within the TME. Western blot analyses of tumor lysates revealed substantial reductions in IDO1, total STAT1, and phosphorylated STAT1 in ConaHA-treated mice (Fig. 5I), consistent with inhibition of IFN-γ–STAT1 signaling in vivo. Multiplex immunofluorescence staining demonstrated increased CD8^+^ T cell infiltration accompanied by decreased KYN, IDO1, IFNGR1, and PD-L1 levels in tumor tissues (Fig. 5J and fig. S6F), indicating restoration of KYN/TRP metabolic balance and relief of local immunosuppression. Enhanced tumor apoptosis was also observed following ConaHA treatment (fig. S6G). Collectively, these results indicate that ConaHA achieves effective tumor targeting, suppresses IFN-γ–STAT1–IDO1 signaling, reprograms the metabolic-immune landscape of the TME, and elicits robust antitumor responses in vivo while maintaining favorable systemic safety.

### ConaHA remodels the tumor immune microenvironment by enhancing CTL antitumor activity

**Fig. 6.**
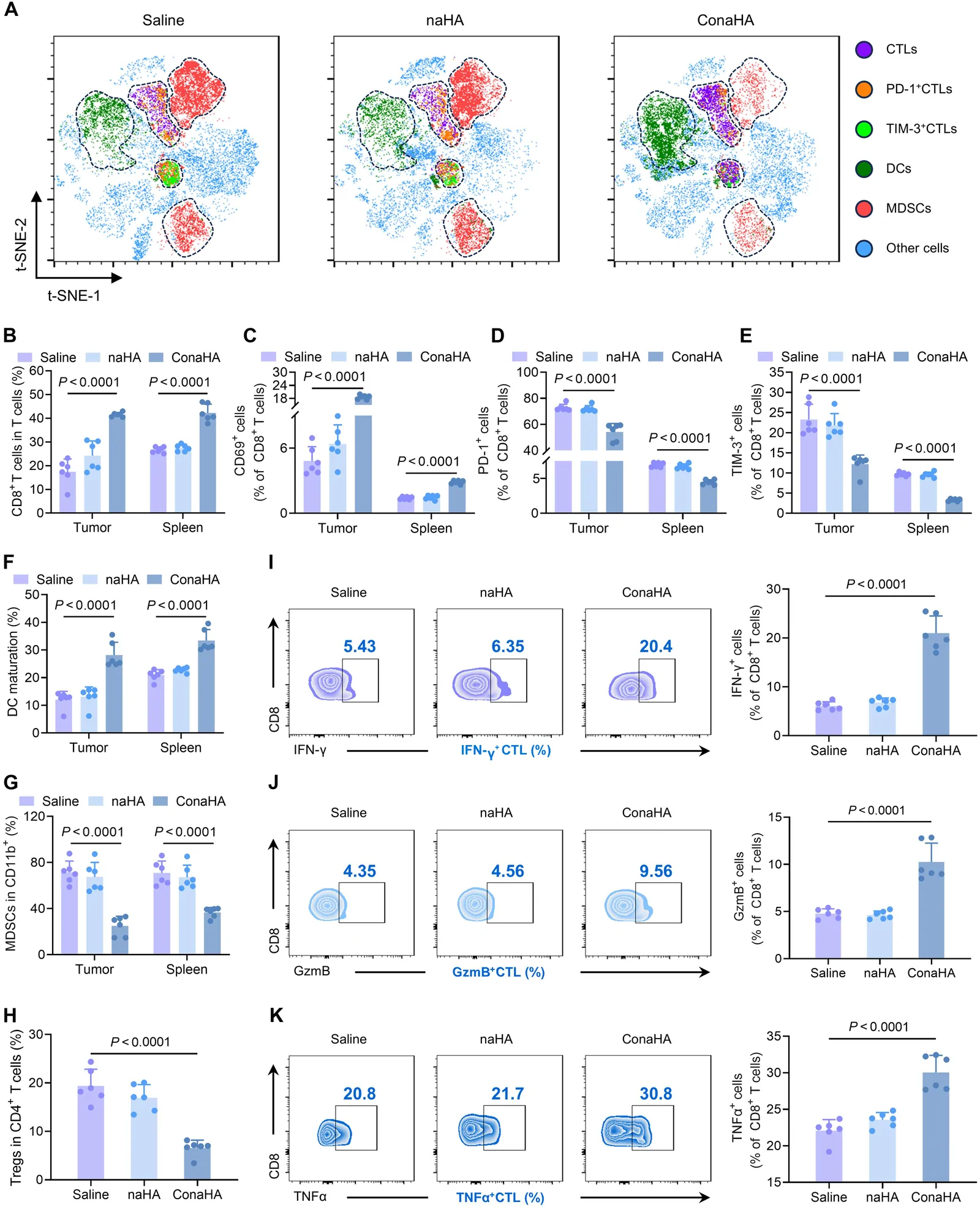
ConaHA remodels the tumor immune microenvironment by enhancing CTL antitumor activity. (**A**) t-SNE analysis of immune cells (CTLs, DCs, and MDSCs) in Panc02 tumors from mice in various groups. (**B** to **G**) Flow cytometry analysis of immune cell frequencies in tumors and spleens of mice from different groups. (B to E) For CD8^+^ T lymphocyte subsets, (F) for mature DCs, and (G) for MDSCs. (**H** to **K**) Flow cytometry analysis of T cell frequencies in tumors. (H) For T_reg_ cells and (I to K) for activated T lymphocyte subsets. Data are presented as mean ± SD (*n* = 6).

The acidic TME and metabolic dysregulation contribute to tumor-associated immunosuppression. To define the immunological impact of ConaHA in vivo, we performed multiparametric flow cytometric profiling of splenic and tumor-infiltrating immune populations in Panc02-bearing mice. The T-SNE analysis of tumor-infiltrating leukocytes revealed pronounced immune remodeling in ConaHA-treated tumors (Fig. 6A). Consistent with its in vivo antitumor efficacy, ConaHA markedly increased CTL infiltration, with CTLs comprising 43.9% of tumor-infiltrating leukocytes and 47.5% of splenic T cells, significantly exceeding levels observed in saline- or naHA-treated controls (Fig. 6B). Meanwhile, ConaHA promoted early-activated CTLs (CD3^+^CD8^+^CD69^+^) while significantly reducing exhausted CTL subsets (CD3^+^CD8^+^PD-1^+^ and CD3^+^CD8^+^TIM-3^+^) in both spleens and tumors, whereas naHA showed negligible effects (Fig. 6, C to E). Given the dependence of CTL priming and memory differentiation on DC maturation, we next assessed antigen-presenting cell phenotypes. ConaHA treatment significantly increased the proportion of mature DCs in tumors (39.0%) compared with naHA (17.1%) and saline controls (14.4%) (Fig. 6F), consistent with enhanced antigen presentation and costimulatory signaling. Concurrently, immunosuppressive myeloid-derived suppressor cells (MDSCs) were markedly reduced to 0.36-fold of saline controls (Fig. 6G), and T_reg_ cells declined to approximately one-third of baseline levels (Fig. 6H), reflecting broad attenuation of suppressive cellular networks.

Given the pronounced CTL infiltration induced by ConaHA within the TME, functionally active T cell subsets were further quantified to comprehensively evaluate its immune activation potential. Compared with the saline group, ConaHA treatment led to a fourfold increase in tumor-infiltrating IFN-γ^+^ CTLs (CD3^+^CD8^+^IFN-γ^+^) and a twofold increase in GzmB^+^ CTLs (CD3^+^CD8^+^GzmB^+^), both of which are key effector populations mediating CTL-dependent antitumor cytotoxicity (Fig. 6, I and J). In addition, the proportion of tumor necrosis factor–α (TNFα)^+^CTLs increased by ∼50% following ConaHA treatment (Fig. 6K). These expansions of effector populations indicate restoration of cytolytic capacity and heightened immune activation. To further clarify whether the antitumor activity of ConaHA depends on immune regulation, we performed additional in vivo experiments using Panc02 tumor–bearing BALB/c nude mice. ConaHA exhibited only limited antitumor efficacy and was insufficient to effectively suppress tumor progression (fig. S7, A to E). This result strongly suggests that its in vivo antitumor activity of Co^2+^ is predominantly mediated through immune modulation.

Collectively, these data demonstrate that ConaHA reshapes the immunosuppressive TME by enhancing DC maturation, limiting suppressive myeloid and T_reg_ cell populations, and promoting activation and functional reinvigoration of CTLs. This coordinated immune remodeling provides a mechanistic basis for the robust antitumor responses observed in vivo.

### ConaHA suppresses tumor progression by attenuating tumor immune suppression

**Fig. 7.**
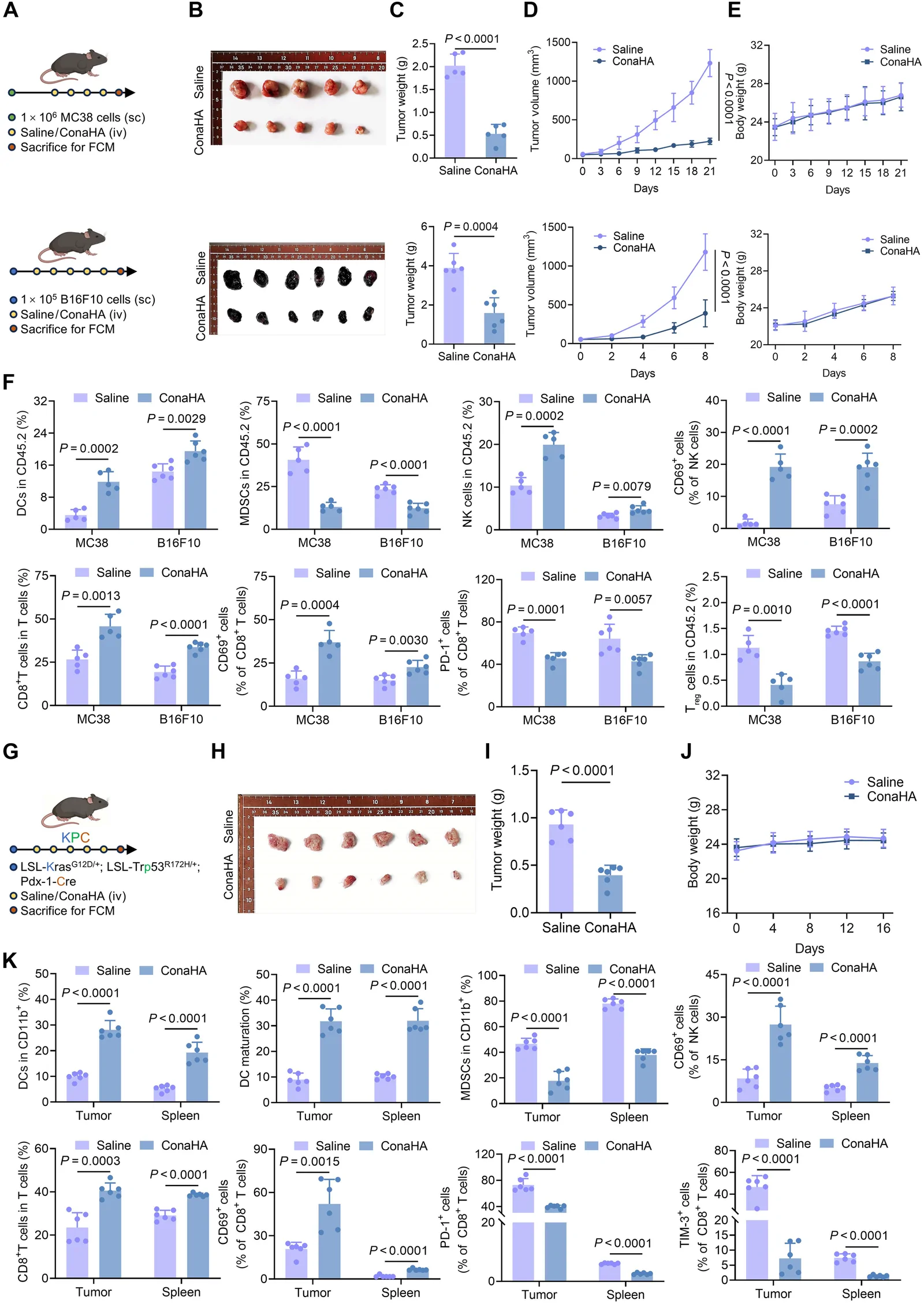
ConaHA suppresses tumor progression by attenuating tumor immune suppression. (**A**) Experimental scheme of the subcutaneous MC38 (top) and B16F10 (bottom) tumor models in male C57BL/6J mice. Saline and ConaHA (40 mg/kg HA) were intravenously (iv) administered via the tail vein after tumor reached 50 mm^3^ (*n* = 5 in the MC38 model, *n* = 6 in the B16F10 model). (**B**) Appearance of tumors in different groups of mice. (**C**) Average tumor weight of various groups (*n* = 5 in the MC38 model, *n* = 6 in the B16F10 model). (**D**) Mean tumor growth curves of mice (*n* = 5 in the MC38 model, *n* = 6 in the B16F10 model) subjected to various treatments. (**E**) Body weight of the mice in the various groups (*n* = 5 in the MC38 model, *n* = 6 in the B16F10 model). (**F**) Flow cytometry analysis of immune cell frequency from tumors (DC: CD45.2^+^ CD11b^+^ CD11c^+^ MHCII^+^, MDSCs: CD45.2^+^ Gr-1^+^ CD11b^+^, NK cells: CD45.2^+^ CD11b^−^ CD3^−^ NK1.1^+^, and T lymphocyte subsets: CD45.2^+^ CD3^+^ CD8^+^ for CD8^+^ T cells, CD45.2^+^ CD3^+^ CD4^+^ CD25^+^ Foxp3^+^ for T_reg_ cells). (**G**) Experimental scheme of orthotopic KPC mice. Saline and ConaHA [cobalt (2.5 mg/kg)] were intravenously administered via the tail vein (*n* = 6). (**H**) Appearance of tumors in different groups of mice (*n* = 6). (**I**) Average tumor weight of various groups (*n* = 6). (**J**) Body weight of the mice in the various groups (*n* = 6). (**K**) Flow cytometry analysis of immune cell frequency from tumors (DC: CD45.2^+^ CD11b^+^ CD11c^+^ MHCII^+^, mature DC: CD45.2^+^ CD11b^+^ CD11c^+^ MHCII^+^ CD86^+^ CD80^+^, MDSCs: CD45.2^+^ Gr-1^+^ CD11b^+^, NK cells: CD45.2^+^ CD11b^−^ CD3^−^ NK1.1^+^, and CD45.2^+^ CD3^+^ CD8^+^ for CD8^+^ T cells) (*n* = 6). Data are presented as mean ± SD. sc, subcutaneous.

To assess the generalizability of ConaHA-mediated metalloimmunotherapy, we extended our evaluation to syngeneic murine models of colon carcinoma and melanoma (Fig. 7A). Consistent with observations in the Panc02 model, systemic administration of ConaHA significantly restrained tumor growth in both settings without affecting body weight, indicating preserved systemic tolerability (Fig. 7, B to E). Flow cytometric profiling of tumor-infiltrating leukocytes revealed pronounced immune remodeling following ConaHA treatment (Fig. 7F). The frequency and absolute number of DCs were markedly increased relative to saline-treated controls, whereas MDSCs were substantially reduce. In parallel, natural killer (NK) cells and CD8^+^ T cells were significantly expanded within the TME, indicating reinforcement of both innate and adaptive cytotoxic compartments. ConaHA reduced the proportion of exhausted CTLs (CD3^+^CD8^+^PD-1^+^) and diminished T_reg_ cell abundance compared with untreated tumors, demonstrating effective alleviation of local immunosuppression. We further adopted the genetically engineered KPC tumor model. The KPC model (*LSL-Kras*^*G12D/+*^; *LSL-Trp53*^*R172H/+*^; *Pdx-1-Cre*) is a well-established spontaneous PDAC model that closely mimics the pathological and immunosuppressive features of human pancreatic cancer. Consistent with the outcomes obtained from subcutaneous tumor models, systemic delivery of ConaHA remarkably suppressed tumor progression in this orthotopic KPC model (Fig. 7, G to J). Moreover, as shown in Fig. 7K, ConaHA treatment markedly facilitated DC maturation, increased the infiltration and proportion of cytotoxic CD8^+^ T cells and NK cells, and meanwhile reduced the abundance of MDSCs in both tumor tissues and spleens of KPC mice. These coordinated changes were accompanied by enhanced tumor control, supporting a mechanistic link between immune reinvigoration and therapeutic efficacy.

Together, these findings establish that ConaHA suppresses tumor progression by alleviating the immune suppression of CD8^+^ T cells and dismantling suppressive cellular networks within the TME. The reproducible antitumor activity across distinct tumor types highlights the broad translational potential of cobalt-based metalloimmunotherapy.

## DISCUSSION

Metal ions play indispensable roles in the regulation of immune signaling and cellular immune functions (*23*). Accumulating evidence has demonstrated that metal ions participate in diverse immunological processes, leading to the emergence of metalloimmunotherapy as a promising therapeutic paradigm (*26*). Metal-based therapeutics have long been used in clinical medicine, and recent advances in understanding the interactions between metal ions and immune-related biomolecules, cells, and pathways have further expanded their potential in immunotherapy. For instance, Mn^2+^ has been extensively reported to activate the cGAS-STING signaling pathway and enhance innate immune responses (*36*, *41*), whereas Cu^2+^-based systems have been shown to induce cancer cell damage and immunogenic cell death. In contrast, the immunomodulatory roles of other metal ions, including Co^2+^, Zn^2+^, and Fe^2+^/Fe^3+^, remain comparatively underexplored. Emerging studies suggest that these metal ions may regulate immune responses through multiple mechanisms, including modulation of innate immune sensing, redox homeostasis, immune metabolism, and TME remodeling. IDO1, the primary enzyme responsible for catalyzing the conversion of TRP to KYN, has been widely recognized as a prognostic marker for immune checkpoint blockade therapy (*14*, *42*). Although IDO1 has emerged as a promising therapeutic target for cancer immunotherapy, clinical trials evaluating IDO1 inhibitors, such as epacadostat, have failed to demonstrate significant clinical benefits (*43*, *44*). These limitations highlight the urgent need for alternative therapeutic strategies capable of modulating the IDO1-mediated immunosuppression through distinct mechanisms, thereby prompting increasing interest in emerging approaches such as metalloimmunotherapy.

In this study, we identified Co^2+^ as a potent suppressor of IFN-γ–induced IDO1 expression across several cancer cell lines through systematic screening of multiple metal ions, accompanied by a marked reduction in KYN production. Mechanistically, Co^2+^ interacted with the His-83 residue of IFNGR1, promoting receptor degradation and consequently attenuating IFN-γ–JAK–STAT1 signaling. These findings identify IFNGR1 as a previously unrecognized molecular target of Co^2+^ and provide a mechanistic framework linking Co^2+^ signaling to the regulation of tumor immunometabolism.

Although IFNGR1 is broadly expressed across multiple cell types and indispensable for IFN-γ–mediated antigen presentation, immune surveillance, and antitumor immunity, Co^2+^ induced markedly different effects on IFNGR1 degradation across distinct cell types. At equivalent concentrations, Co^2+^ preferentially promoted IFNGR1 degradation in tumor cells while largely preserving receptor expression in immune cells. This selective vulnerability attenuated tumor-intrinsic IFN-γ signaling, reprogrammed the KYN-TRP metabolic axis, relieved immunosuppression within the TME, and enhanced antitumor immunity. The basis for this differential sensitivity remains to be fully defined but may reflect cell type–specific differences in Co^2+^ uptake, intracellular metal homeostasis, or the molecular machinery governing IFNGR1 turnover. Such selectivity may provide a therapeutic window in which tumor-intrinsic IFN-γ signaling is preferentially suppressed without compromising immune cell function. Nevertheless, the clinical application of free Co^2+^ is constrained by systemic toxicity and limited tumor selectivity, underscoring the need for targeted delivery strategies.

To overcome these limitations, we engineered ConaHA, a HA-based tumor-targeted nanoplatform capable of sustained and controlled Co^2+^ release. This nanoplatform enhanced intratumoral accumulation while minimizing off-target adverse effects, thereby notably improving therapeutic efficacy and biosafety. Collectively, we explored a cobalt-based metalloimmunotherapeutic strategy to modulate tumor immunosuppression, thereby demonstrating the feasibility of integrating metal ion immunoregulation with nanotechnology-based delivery systems for precision cancer immunotherapy and highlighting the emerging potential of metalloimmunology as a rapidly evolving field in cancer therapy.

In summary, our study demonstrates that metal ion signaling can be exploited to selectively modulate immune regulatory pathways within tumors. By identifying IFNGR1 as a previously unrecognized target of Co^2+^, we uncover a cell type–specific vulnerability that enables preferential inhibition of tumor-intrinsic IFN-γ signaling while preserving immune competence. These findings establish a mechanistic rationale for developing tumor-targeted Co^2+^ delivery platforms to enhance therapeutic specificity, minimize systemic toxicity, and facilitate the translation of Co^2+^-based immunometabolic therapies.

## MATERIALS AND METHODS

### Reagents and antibodies

HA (9004-61-9), 2-nitroimidazole (527-73-1), and 6-(Boc-amino) hexyl bromide (142356-33-0) were purchased from Bidepharm (China). CoCl_2_·6H_2_O (7791-13-1) was purchased from Sigma-Aldrich. IDO1 (D8W5E), PD-L1 (D4H1Z), JAK2 (D2E12), Phospho-JAK2 (Tyr^1007/1008^), and HA-Tag (C29F4) were purchased from Cell Signaling Technology. IFNGR1/CD119 polyclonal antibody, LaminB1 polyclonal antibody, and glyceraldehyde phosphate dehydrogenase monoclonal antibody (mAb) were purchased from Proteintech. STAT1 and Phospho-STAT1 were purchased from Selleck. KYN antibody was purchased from Santa Cruz Biotechnology. JAK1 (PT0658R) PT rabbit mAb, JAK1 (Phospho Tyr^1034/1035^) (PT0991R) PT rabbit mAb, and IFNGR1 (PT1845R) PT rabbit mAb were purchased from Immunoway. All antibody usage concentrations were officially recommended concentrations. The CellXViva Mouse T cell Activation Kit was purchased from STARTER. BD IMag Anti-Mouse CD8a Particles-DM was purchased from BD (USA). The CD4^+^ T Cell Isolation Kit-mouse was purchased from Miltenyi Biotec.

### Cell lines

Murine tumor cell lines Panc02, KPC, MC38, and B16F10 and normal murine cell lines L929 and RAW264.7 were cultured in Dulbecco’s modified Eagle’s medium (DMEM) containing 10% fetal bovine serum (FBS) and 1% penicillin-streptomycin solution at 37°C under 5% CO_2_.

### Western blot

Panc02 cells were seeded in 24-well plates at a density of 5 × 10^4^ cells per well and treated with saline or various concentrations of cobalt compounds for 24 hours in the presence or absence of IFN-γ (final concentration at 50 ng/ml). Following treatment, cell lysates were diluted in 1× sample buffer and boiled in a metal bath at 100°C for 15 min. Samples were loaded onto 10% SDS–polyacrylamide gel electrophoresis (SDS-PAGE) gels for separation and subsequently electro-transferred onto 0.45-μm nitrocellulose membranes. Membranes were blocked with 5% nonfat milk diluted in TBST for 1 hour at room temperature and then cut according to molecular weight (MW) markers and incubated with primary antibodies at 4°C overnight. After incubation, membranes were washed three times with TBST (5 min per wash) and then incubated with horseradish peroxidase (HRP)–conjugated secondary antibodies at room temperature for 1 hour. Following three additional TBST washes, protein bands were visualized using HRP substrate and imaged with a GE Amersham Imager 300 system.

### Ion screening

Panc02 cells were seeded in 24-well plates at a density of 5 × 10^4^ cells per well and treated with saline or different metal ions (Co^2+^, Mg^2+^, Fe^2+^, Ca^2+^, Cu^2+^, Ni^2+^, and Mn^2+^) at 200 μM under IFN-γ stimulation (50 ng/ml) for 24 hours. Then, IDO1 protein level was analyzed by Western blot to evaluate the inhibitory ability of various metal ions on IDO1.

### Enzyme linked immunosorbent assay

Serum or supernatants of cell culture were assayed for mouse KYN (USCN, UCEX300Ge), mouse IFN-γ (R&D Systems, DY485), and GzmB (R&D Systems, DY1865) according to the manufacturer’s instructions. Biotek Cytation5 was used to detect the signals at 450 nm.

### Quantitative RT-PCR

Panc02 cells were seeded in 60-mm dishes at a density of 1 × 10^5^ cells per dish and treated with saline or cobalt compounds in the presence or absence of IFN-γ for 24 hours. Total RNA was extracted from the treated cells using RNAiso Plus reagent (TaKaRa, catalog no. 9108) according to the manufacturer’s protocol. The mRNA expression levels were determined using FastStart Essential DNA Green Master (Roche) on a LightCycler 96 System (Roche), following the manufacturer’s instructions. Relative mRNA expression levels were normalized and analyzed using the 2^−^ΔΔCt method. The sequences of the primers used in this study are listed in table S2.

### Immunofluorescence

The Panc02 cells were seeded in 35-mm confocal dishes (5 × 10^4^ cells per dish) and treated with saline or cobalt compounds in the presence or absence of IFN-γ for 24 hours. After treatment, the cells were rinsed with phosphate-buffered saline (PBS) three times, permeabilized with 0.3% Triton X-100 in PBS for 5 min, and blocked by 5% bovine serum albumin in PBS at room temperature for 0.5 hours. After overnight incubation with STAT1 antibody (1:500, Selleck, F0263) at 4°C, the coverslips were washed three times with PBST and incubated with a fluorescent secondary antibody (1:800, Alexa Fluor 488 A-11008) for 1 hour in the dark at room temperature. The coverslips were washed three times with PBST and incubated with 4′,6-diamidino-2-phenylindole (1 μg/ml) for 10 min, and then the coverslips were washed five times with PBST and mounted with PBS. Images were acquired at room temperature using a confocal microscope (LSM 980-NLO, Zeiss).

### T cell isolation and activation

Mouse CD4^+^ T cell and CD8^+^ T cells were separated from C57BL/6J mouse spleen with a CD4^+^ T cell or CD8^+^ T cell enrichment kit. CD8^+^ T cells were stimulated and cultured with plate-bound anti-CD3 (5 μg/ml) and anti-CD28 (5 μg/ml) and fully supplemented tissue culture medium [RPMI plus 10% FBS, 25 mM Hepes, 5 μM mercaptoethanol, 1% penicillin-streptomycin, and interleukin-2 (IL-2; 10 μg/ml)] for 3 days.

### T cell–mediated tumor cell killing assay

Panc02 cells were first treated with saline or cobalt compounds in the presence or absence of IFN-γ for 24 hours. Subsequently, the treated Panc02 cells were cocultured with activated mouse CD8^+^ T cells at an effector-to-target ratio of 8:1 for 2 days. After coculture, Panc02 cells were harvested, stained with the Annexin V–FITC Apoptosis Detection Kit (catalog no. C1062S, Beyotime, China) and then analyzed by flow cytometry (BD Symphony A1) to assess cell apoptosis.

### In vitro coculture assay

Panc02 cells treated with saline or cobalt compounds in the presence of IFN-γ for 24 hours were cocultured with activated mouse CD4^+^ T cells or CD8^+^ T cells at the T:E ratio of 1:2 for 3 days, and then CD4^+^ T cells or CD8^+^ T cells were harvested and stained to analyze the percentage of CD25^+^Foxp3^+^CD4^+^T cells or PD-1^+^CD8^+^ T cells and TIM-3^+^ CD8^+^ T cells by flow cytometry.

Immature BMDCs isolated from femurs and tibias of C57BL/6J mice were cultured in RPMI 1640 medium containing 10% FBS, 1% penicillin-streptomycin solution, granulocyte-macrophage colony-stimulating factor (20 ng/ml), and IL-4 (10 ng/ml) for 6 days. DC coculture assay was conducted as immature BMDCs cocultured with same treated Panc02 cells at the ratio of 1:1 for 1 day, and then the DCs were harvested and stained to analyze the percentage of CD80^+^CD86^+^ DCs via BD Symphony A1. Gating strategies are provided in fig. S9.

### Apoptosis assay

The Panc02 cells were seeded in six-well plates (5 × 10^5^ cells per well) and treated with saline or cobalt compounds for 24 hours. The cells were harvested and performed with the Annexin V–FITC Apoptosis Detection Kit (catalog no. C1062S, Beyotime, China) according to the manufacturer’s instructions. Subsequently, the cells were analyzed by a flow cytometer (BD Symphony A1).

### RNA sequencing

Total RNA was extracted from Panc02 cells treated with saline or Co^2+^ ± IFN-γ. RNA integrity was checked using an Agilent Bioanalyzer 2100. Poly(A) mRNA was purified by poly-T magnetic beads and fragmented. First- and second-strand cDNA were synthesized, followed by end repair, A-tailing, adapter ligation, size selection (370 to 420 bp), and PCR amplification. Library quality was validated by Qubit, quantitative PCR, and Bioanalyzer. Libraries were clustered and sequenced on an Illumina NovaSeq platform to generate 150-bp paired-end reads.

Raw reads were filtered by fastp to remove adapters and low-quality reads. Clean reads were aligned to the reference genome using Hisat2 v2.0.5. Gene expression was quantified by featureCounts and normalized to FPKM. Differential expression analysis was performed using the DESeq2 R package. *P* values were adjusted via the Benjamini-Hochberg method to control FDR; genes with adjusted *P* ≤ 0.05 were defined as DEGs. GO and KEGG enrichment analyses were conducted using clusterProfiler with correction for gene length bias.

### Cell fractionation

Panc02 cells treated with saline or cobalt compounds (in the presence or absence of IFN-γ) were washed with cold PBS and harvested. Cytoplasmic and nuclear fractions were then isolated using a Nuclear and Cytoplasmic Protein Extraction Kit (Beyotime, Shanghai, China, catalog no. P0028) according to the manufacturer’s instructions. Protein concentrations of the fractions were determined using a BCA protein assay kit. Equal amounts of protein from each fraction were subsequently subjected to Western blot analysis as described above.

### Plasmid construction and transfection

The pLV3-CMV-IFNGR-3×HA-Puro plasmid was obtained from MiaoLingPlasmid (China). All newly generated mutant plasmids were derived from the wild-type pLV3-CMV-IFNGR1-3×HA-Puro construct (table S3). To generate the H83A and H461A point mutants, homologous recombination-based seamless cloning was performed using a commercial kit (Vazyme). The pLV3-CMV-IFNGR1-3×HA-Puro plasmid was linearized by inverse PCR or restriction enzyme digestion. The mutant inserts were amplified using mutagenic primers (table S4) containing 15- to 20-bp homology arms to the linearized vector. The linearized vector and purified PCR products were assembled via homologous recombination according to the manufacturer’s instructions. All constructs were confirmed by Sanger sequencing. The complete sequences of the plasmids are available at (GenBank/Addgene) under the accession numbers listed in “Data, code, and materials availability” statement.

### In vitro pull-down assay

Human embryonic kidney (HEK) 293T cells were transfected with 9 μg of IFNGR1-WT, IFNGR1-Mut83, or IFNGR1-Mut461 plasmids via PEI. Twenty-four hours later, cells were lysed in 1% Triton lysis buffer, and protein concentration was determined. Aliquots of lysates were retained as input controls. Lysates with 60 μg of total protein were incubated with Co^2+^-chelating magnetic beads (Co NTA Beads) overnight at 4°C for His-tagged IFNGR1 enrichment. After washing to remove nonspecific binding, beads were boiled in SDS-PAGE sample buffer for Western blot detection.

### Synthesis of naHA and ConaHA

aHA was prepared as previously reported with minor modifications (*45*). Briefly, HA (1 g; MW, 200 kDa) was fully dissolved in triple-distilled water (TDW) to a final concentration of 10 mg/ml. A sodium periodate (NaIO_4_; Sigma-Aldrich, St. Louis, MO, USA) solution was added dropwise to the HA solution at an equimolar ratio relative to HA, followed by stirring at room temperature in the dark for 2 hours. Subsequently, 1 ml of ethylene glycol (Sigma-Aldrich) was added, and the mixture was stirred in the dark for an additional 1 hour to quench unreacted NaIO_4_. The reaction solution was purified by dialysis against TDW using a 2-kDa MW cutoff dialysis membrane (Membrane Filtration Products Inc., Seguin, TX, USA) for 3 days. The purified solution was then lyophilized and stored at 4°C until use.

NI-NH_2_ was synthesized on the basis of the method described previously (*46*). Briefly, 2-NI (0.302 g, 12.67 mmol) was dissolved in 10 ml of *N*,*N*-dimethylformamide, followed by the addition of K_2_CO_3_ (1.114 g, 4.06 mmol). 6-(Boc-amino) hexyl bromide (0.782 g, 2.8 mmol) was then added dropwise, and the mixture was stirred at 80°C for 12 hours. After cooling, 10 ml of methanol was added to the reaction mixture, which was subsequently extracted with ethyl acetate three times. The organic layer was dried over anhydrous sodium sulfate and concentrated to yield a yellow product. This product was dissolved in 1.8 mL of dichloromethane (CH_2_Cl_2_), and 3.6 ml of trifluoroacetic acid was added. The mixture was stirred at room temperature for 4 hours. The solvent was evaporated under reduced pressure, and the residue was washed to obtain amine-functionalized 2-NI (NI-NH_2_) as a yellow oil.

For the synthesis of naHA, aHA (0.5 g, 0.63 mmol) was dissolved in 0.1 M MES buffer (pH 5.5). 1-Ethyl-3-(3-dimethylaminopropyl)carbodiimide (0.182 g, 0.95 mmol) and *N*-hydroxysuccinimide (0.146 g, 1.27 mmol) were added, and the mixture was stirred for 15 min. NI-NH_2_ (0.315 g, 1.27 mmol) was then slowly added to the reaction mixture, which was stirred for 24 hours at room temperature. The resulting solution was dialyzed against distilled water using a 2-kDa MW cutoff membrane for 2 days and subsequently lyophilized. The successful grafting of NI onto aHA was confirmed by ^1^H nuclear magnetic resonance spectroscopy.

To prepare the naHA-Co^2+^ complex, naHA (0.5 g) was dissolved in 30 ml of distilled water. A solution of CoCl_2_·6H_2_O (0.2 g) in 1 ml of distilled water was added dropwise to the naHA solution, followed by stirring for 24 hours at room temperature. The resulting solution was dialyzed against distilled water using a 500-Da MW cutoff membrane for 2 days and then lyophilized.

### Characterization of ConaHA NPs

The hydrodynamic size distribution and zeta potential were measured by a Nano ZSE Zetasizer (Malven, UK). Scanning electron microscope was conducted on Crossbeam 330 (ZEISS, German) to characterize the morphology and composition of ConaHA. XPS measurement was obtained from an Escalab Xi+ x-ray photoelectron spectrometer (Thermo Fisher Scientific, UK). The metal element contents of cobalt were determined by an iCAP RQ (Thermo Fisher Scientific, USA) inductively coupled plasma−mass spectrometry (ICP-MS).

### In vitro cobalt release assay

ConaHA NPs were diluted to a cobalt-equivalent concentration of 24 μg/ml in 4 ml of either 0.01 M PBS (pH 7.4) or 0.01 M PBS (pH 6.5), followed by incubation at 37°C for different time intervals. After incubation, samples were centrifuged at 14,000*g* and 4°C for 40 min. The cobalt content in the supernatant, representing cobalt released from degraded nanoparticles, was quantified using ICP-MS.

### MTT assay

Various mouse cell lines were respectively seeded in 96-well plates at a density of 1 × 10^4^ cells per well and treated with saline or various concentrations of ConaHA NPs for 24 hours. MTT reagent (Bio Basic, catalog no. T0793-500 MG) was then added to each well to a final concentration for 0.5 mg/ml, followed by incubation at 37°C for 4 hours. After incubation, the formazan crystals formed were resuspended in 150 μl of dimethyl sulfoxide. The absorbance of each well at 492 nm was measured using a Biotek Cytation5 microplate reader.

### Cell migration assay

The Panc02 orB16F10 cells were cultured in a 24-well plate, and a wounded was created by manually scraping with a p10 pipet tip and washed with 1 ml of PBS. The images were acquired when the cells were treated with saline or ConaHA and its components at 0, 6, 12, and 24 hours. The images were compared to quantify the migration rate of the cells.

### Colony formation assay

Panc02 or B16F10 cells were seeded (500 per well) on a six-well plate and treated with saline or ConaHA and its components for 24 hours and then changed to DMEM medium with 10% FBS. After being cultured for another 7 days, colonies were fixed with 4% paraformaldehyde for 15 min and then washed with PBS for three times and stained with 0.1% of crystal violet and imaged.

### Histological analysis

Heart, liver, spleen, lung, kidney, and tumor tissues were fixed with 10% neutral buffered formalin for 48 hours, followed by dehydration and paraffin embedding. Sections (4 μm) were used for staining with hematoxylin and eosin. Whole-slide images were analyzed using CaseViewer software.

### In vivo distribution

The mice were injected via the tail vein with Cy7-labeled ConaHA NPs [with the dose based on cobalt (2.5 mg/kg)]. At different times (0.5, 6, 12, 24, and 48 hours), the mice were anesthetized and imaged using an In-Vivo Xtreme imaging system (Bruker Corporation). Subsequently, the mice were euthanized, and major organs (heart, liver, spleen, lung, kidney, and tumor) were collected followed by fluorescence imaging with the In-Vivo Xtreme system.

### Establishment of tumor models

All animal experiments were approved by the Animal Center of South China University of Technology (2019015). All animals received care in compliance with the guidelines outlined in the Guide for the Care and Use of Laboratory Animals and were maintained at the specific pathogen–free animal facility at the South China University of Technology. C57BL/6J mice and BALB/c-nude mice (male, 6 to 8 weeks old) were purchased from GemPharmatech (Nanjing, China). For tumor model establishment, C57BL/6J mice were subcutaneously inoculated in the right flank with 1 × 10^6^ Panc02 cells, 1 × 10^6^ MC38 cells, or 5 × 10^5^ B16F10 cells suspended in 100 μl of PBS, generating the Panc02, MC38, and B16F10 tumor models, respectively. In addition, BALB/c nude mice were subcutaneously injected in the right flank with 1 × 10^6^ Panc02 cells in 100 μl of PBS to establish the immunodeficient Panc02 model. Mice were randomly divided into two or three groups (six mice for each group): PBS, naHA, and ConaHA [with the dose based on cobalt (2.5 mg/kg)]. The mice were monitored and administered medication by tail intravenous injection on day 7. The treatments were performed every other day. Subcutaneous tumor volume was measured using calipers and estimated using the following formula: Tumor volume (mm^3^) = (width)^2^ × length × 1/2. All mice were euthanized, and all subcutaneous tumors were excised and weighed. Subcutaneous tumor tissues were harvested for subsequent immunophenotypic analysis.

The KPC (*LSL-Kras^G12D/+^*; *LSL-Trp53^R172H/+^*; *Pdx-1-Cre*, 10 weeks old) transgenic mice were purchased from MODEL ORGANISMS Inc. (Shanghai, China). The mice were randomized divided into saline and ConaHA groups (*n* = 6 per group), ConaHA was administered at a cobalt-equivalent dose of 2.5 mg/kg, and all groups received identical dosing regimens and administration schedules, injected via the tail vein every 5 days for a total of five times. Mice were euthanized, and tumor tissues were harvested for subsequent immunophenotypic analysis on day 16.

### Analysis of immune cells

After in vivo treatment, mice were euthanized by eyeball enucleation, and blood samples were collected. Tumors and spleens were harvested for single-cell suspension preparation. Single-cell suspensions were generated from either cultured cells or tumors of tumor-bearing mice. For tumor samples from mice, single-cell suspensions were prepared via sequential steps: rapid and gentle dissection, physical grinding at 4°C, enzymatic digestion with collagenase IV at 37°C for 1 hour, and filtration through a cell strainer. The resulting cell suspension was resuspended in 40% Percoll reagent and centrifuged to enrich immune cells in the pellet. Red blood cells in the pellet were lysed with ACK lysis buffer at room temperature for 5 min, followed by PBS washing. The enriched immune cells were then counted.

For flow cytometric immunophenotyping, enriched immune cells were blocked with CD16/CD32 antibody at 4°C for 20 min to prevent nonspecific antibody binding and subsequently stained with the indicated fluorochrome-conjugated antibodies at 4°C for 30 min. For intracellular staining of IFN-γ, TNFα, Foxp3, and GzmB, cells were permeabilized using a Cytofix/Cytoperm Kit (catalog no. 00-5523-00, Thermo Fisher Scientific) before antibody incubation, following the manufacturer’s instructions. All flow cytometry data were acquired using a BD Symphony A1 flow cytometer and analyzed with FlowJo software (v10.8.1). Gating strategies are provided in fig. S9. The antibodies used in this study are listed in table S1.

### Fluorescent multiplex immunohistochemistry

For immunofluorescence staining of paraffin-embedded tumor tissue sections, sections were first dewaxed and rehydrated through a graded series of xylene and ethanol solutions. After blocking with 10% low endotoxin serum (Beyotime, catalog no. C0265), sections were incubated with primary antibodies against IDO1, KYN, and CD8a for 1 hour at room temperature. Subsequently, sections were stained using the TSA Fluorescence System Kit (PANOVUE, catalog no. TSA-Rab-275) according to the manufacturer’s instructions. Fluorescent images were acquired at room temperature using a confocal laser scanning microscope.

### Statistical analysis

For statistical comparisons among more than two experimental groups, one-way or two-way analysis of variance (ANOVA) was performed, followed by post hoc multiple comparisons tests with correction for multiple testing. Longitudinal tumor growth data with repeated measurements over time were analyzed using repeated-measures ANOVA. Survival outcomes were evaluated using Kaplan-Meier survival analysis, with intergroup differences assessed by the log-rank test. In addition, all data presentation formats were standardized, and data are consistently presented as mean ± SD throughout here in Materials and Methods and figure legends. *P* > 0.05 (not significant), *P ≤ 0.05, **P ≤ 0.01, and ***P ≤ 0.001.
