# Supplementary material for: Cobalt-mediated suppression of IFN-γ–JAK–STAT1 signaling reprograms IDO1-driven immunosuppression for metalloimmunotherapy
Source: Sci Adv. 2026 Sep 18;12(38):eaeg6398. doi: 10.1126/sciadv.aeg6398 (PMC13588167; doi:10.1126/sciadv.aeg6398)
Supplement: Supplementary file 1 [file sciadv.aeg6398_sm.pdf]

Science Advances
AAAS

Supplementary Materials for

Cobalt-mediated suppression of IFN-γ–JAK–STAT1 signaling reprograms IDO1-driven immunosuppression for metalloimmunotherapy

Tianyi Jiang *et al.*

Corresponding author: Yunjiao Zhang, zhangyunjiao@scut.edu.cn

Sci. Adv. **12**, eaeg6398 (2026)
DOI: 10.1126/sciadv.aeg6398

This PDF file includes:

Figs. S1 to S9
Tables S1 to S4

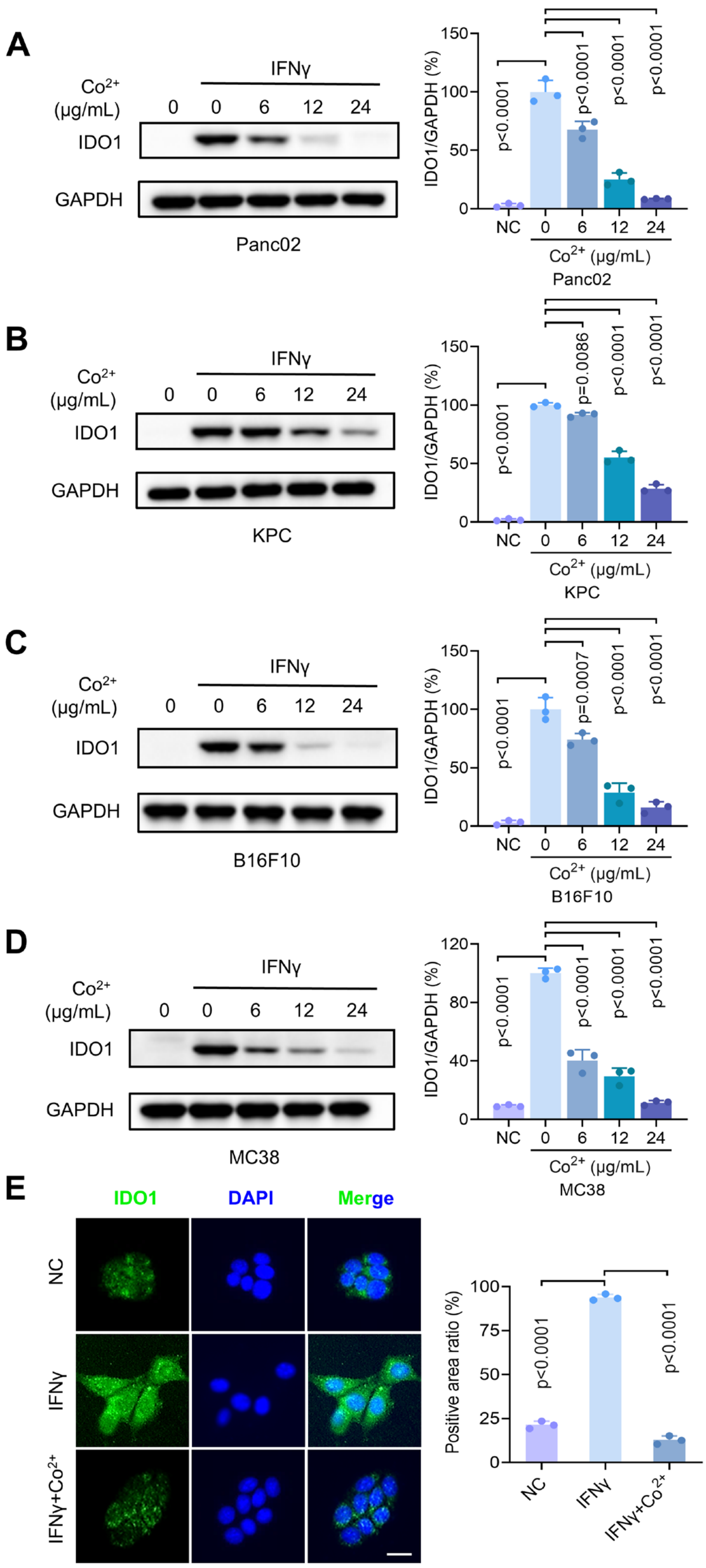

fig. S1. Co²⁺ Downregulates IFNγ-Induced IDO1. A-D) Western blot and quantitative analysis of the expression of IDO1 in PBS or Co^{2+}-treated Panc02, KPC, B16F10 and MC38 cells upon IFNγ stimulation. **E)** Immunofluorescence of Panc02 cells treated with Co^{2+} or PBS upon IFNγ stimulation, Alexa Fluor 488 was used to label IDO1 and DAPI for nucleus (Scale bar: 20 μm). Data are presented as mean ± SD (n = 3).

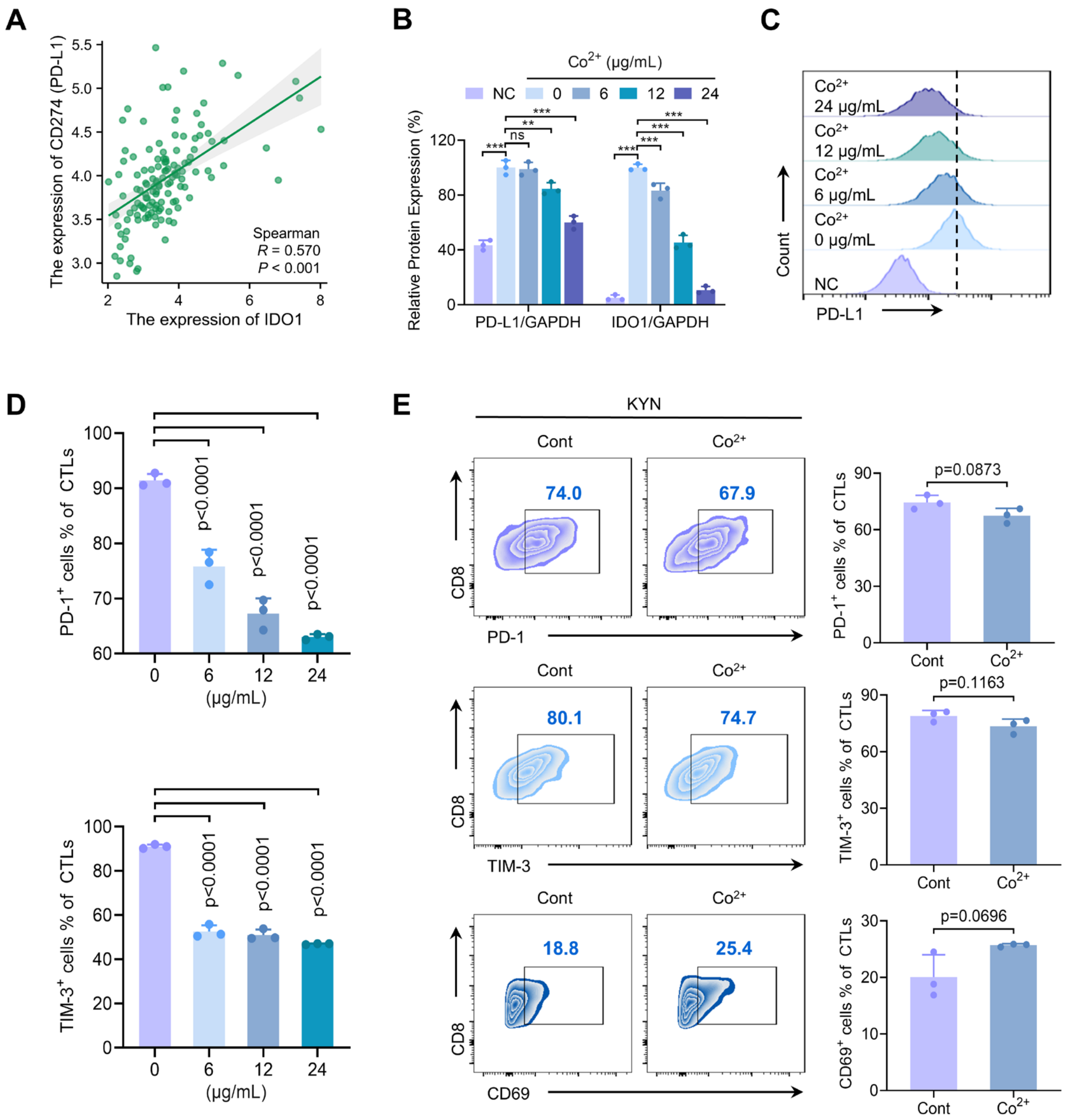

fig. S2. Co²⁺ Alleviates IDO1/PD-L1-Driven Immune Suppression. A) Correlation analysis of PD-L1 and IDO1 expression in the GSE62452 dataset. **B)** Western blot quantitative analysis of the expression of IDO1 in Co^{2+} or PBS treated Panc02 cells upon IFNγ stimulation. **C)** Flow cytometry analysis of PD-L1 levels in Panc02 cells treated with Co^{2+} (0, 6, 12, 24 μg/mL) under IFNγ stimulation. PBS treated cells without IFNγ stimulation served as the negative control (NC). **D)** Quantitative analysis of PD-1^+ CTLs, TIM-3^+ CTLs after indirect co-culture with IFNγ- and Co^{2+}-treated Panc02 cells for 72 h. **E)** Flow cytometry and quantitative analysis of PD-1^+ CTLs, TIM-3^+CTLs and $CD69^+$CTLs after indirect co-culture with IFNγ- and Co^{2+}-treated Panc02 cells in the presence of kynurenine (KYN) for 72 h. Data are presented as mean ± SD (n = 3).

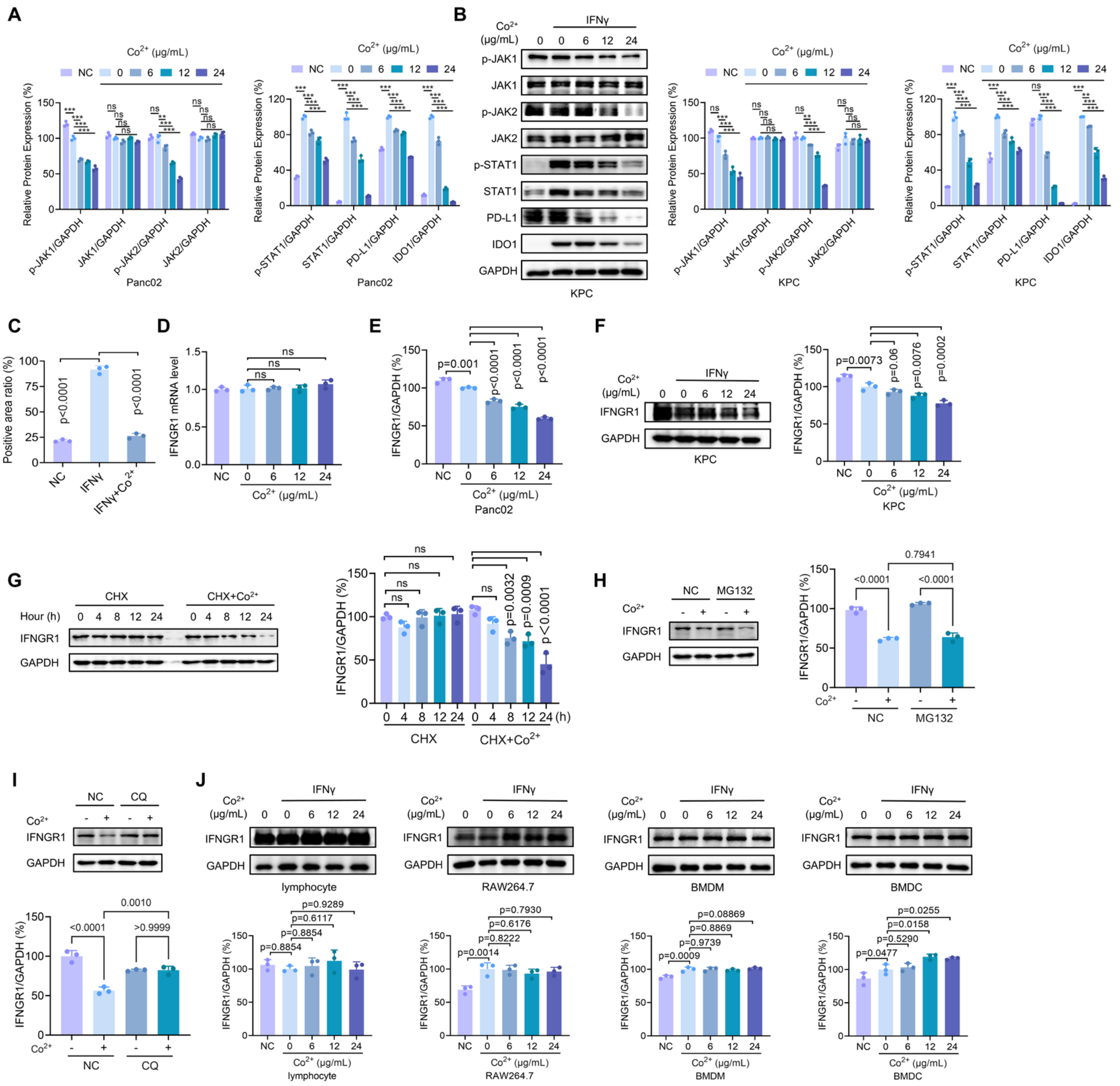

fig. S3. Co^{2+} Inhibits JAK/STAT Signaling by Binding IFNGR1 and Promoting Its Degradation. A) Quantitative analysis of the indicated proteins in Panc02 cells treated with Co^{2+} (0, 6, 12, 24 μg/mL) upon IFNγ stimulation related to Fig. 3G. **B)** Western blot and quantitative analyses of the indicated proteins in KPC cells treated with Co^{2+} (0, 6, 12, 24 μg/mL) upon IFNγ stimulation. **C)** Quantitative analysis of immunofluorescence staining for STAT1 in IFNγ- and IFNγ + Co^{2+}-treated Panc02 cells with IFNγ stimulation. **D)** RT-qPCR for relative IFNGR1 mRNA level in Panc02 cells treated with Co^{2+} (0, 6, 12, 24 μg/mL) upon IFNγ stimulation. **E)** Quantitative analyses of IFNGR1 in Panc02 cells treated with Co^{2+} (0, 6, 12, 24 μg/mL) upon IFNγ stimulation related to Fig. 3K. **F)** Western blot and quantitative analysis of IFNGR1 in KPC cells treated with Co^{2+} (0, 6, 12, 24 μg/mL) upon IFNγ stimulation. **G)** Western blot and quantitative analysis of IFNGR1 in Panc02 cells treated with cycloheximide

(CHX, 20 μM) either in the absence or presence of Co^{2+} (24 μg/mL) for the indicated hours. **H)** Western blot and quantitative analysis of IFNGR1 in Panc02 cells after the indicated treatment for 24 h. Dosing: Co^{2+}, 24 μg/mL; MG-132, 2.5 μM. **I)** Western blot and quantitative analysis of IFNGR1 in Panc02 cells after the indicated treatment for 24 h. Dosing: Co^{2+}, 24 μg/mL; CQ, 50 μM. **J)** Western blot and quantitative analysis of IFNGR1 protein level in lymphocytes isolated from the spleen of C57BL/6J mice, RAW264.7, BMDM and BMDC treated with Co^{2+} (0, 6, 12, 24 μg/mL) upon IFNγ stimulation. Data are presented as mean ± SD (n = 3).

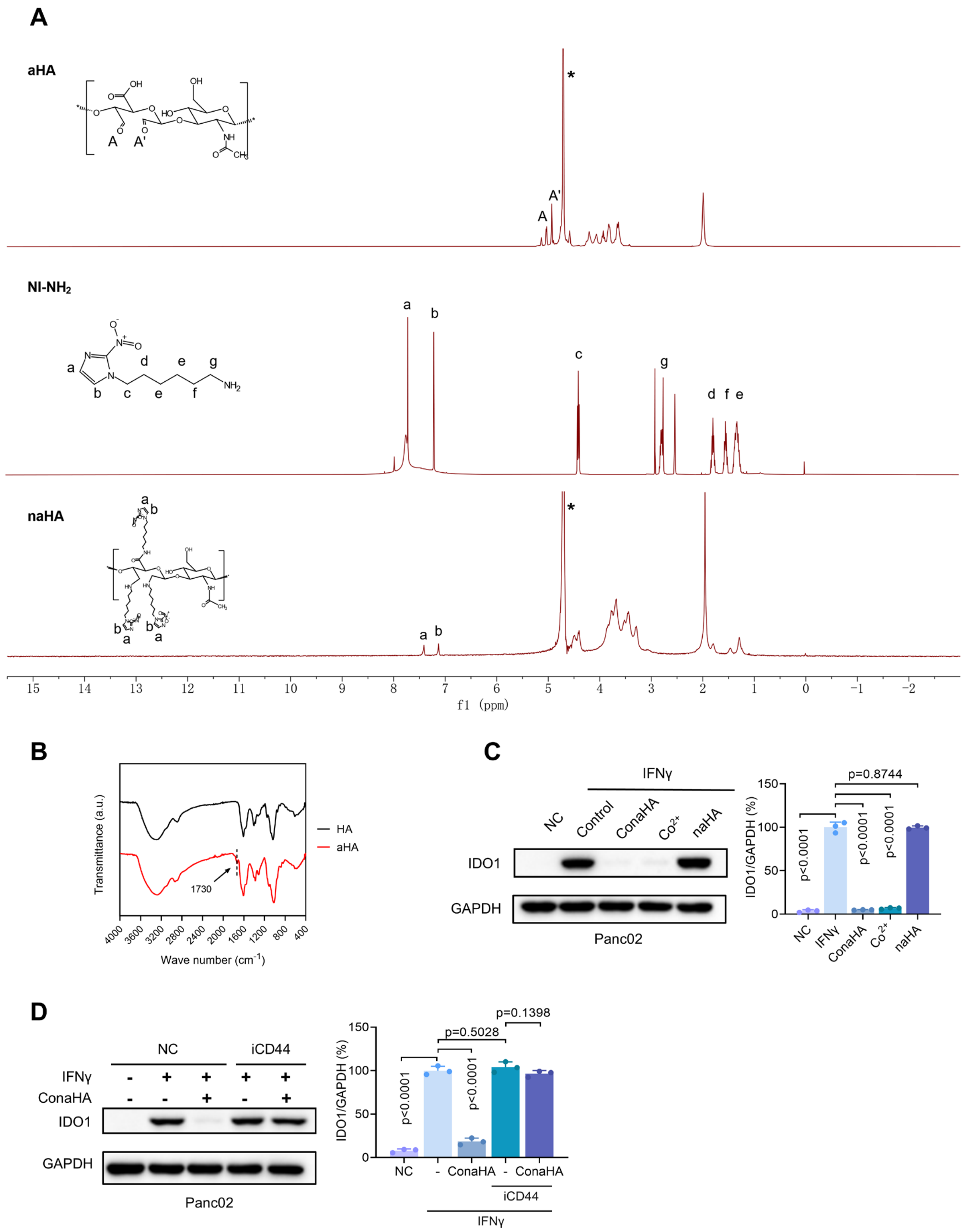

fig. S4. Synthesis and Characterization of ConaHA. A) H-NMR spectrum of aHA, $NI\text{-}NH_2$ and naHA. **B)** Fourier transform infrared (FTIR) spectroscopy of HA and modified aHA. **C)** Western blot and quantitative analysis of the expression of IDO1 in Panc02 cells upon IFNγ stimulation. **D)** Western blot and quantitative analysis of the expression of IDO1 in ConaHA- or PBS- treated Panc02 cells upon IFNγ stimulation with or without CD44 inhibitor. Data are presented as mean ± SD (n = 3).

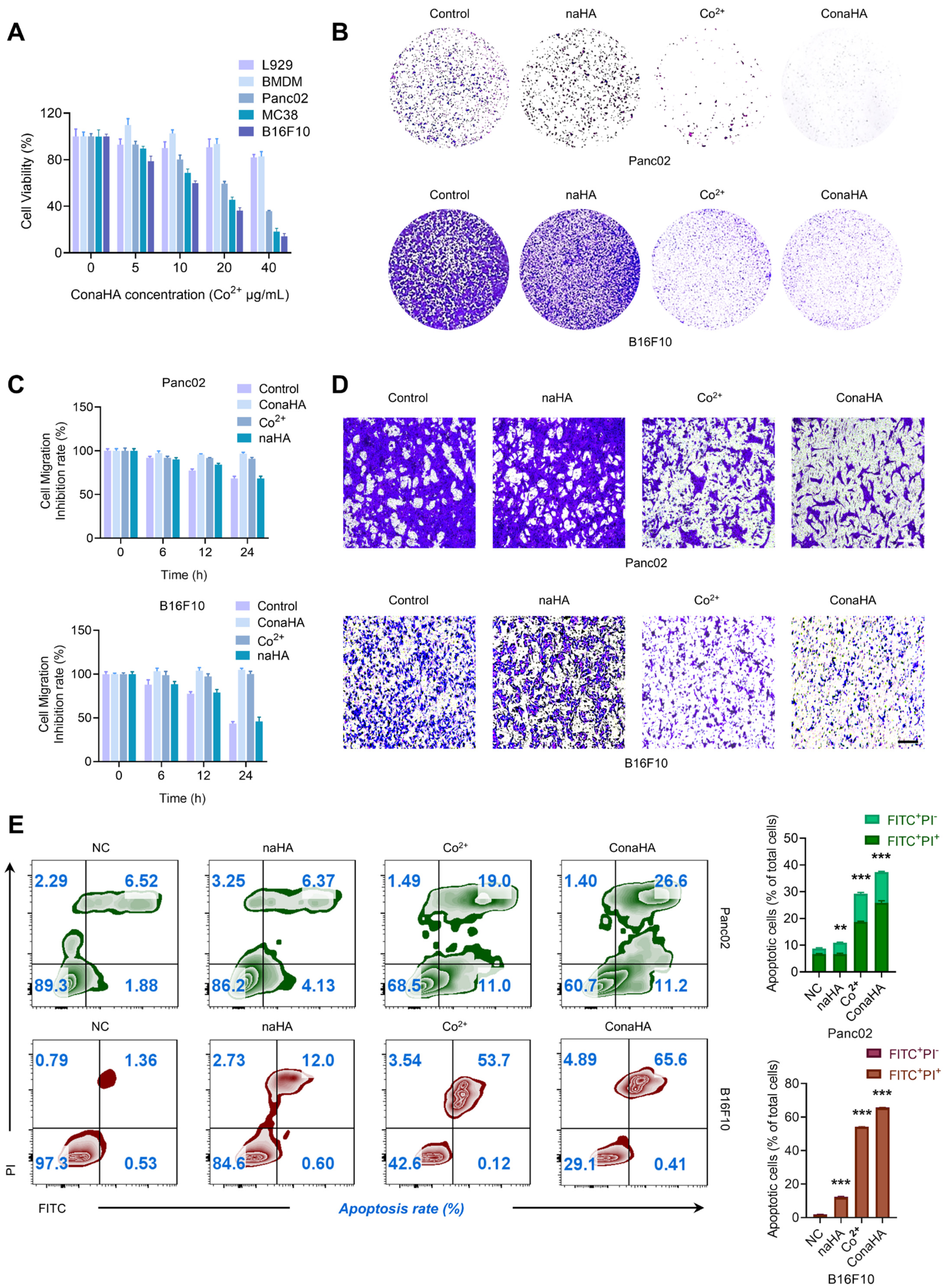

fig. S5. ConaHA Exhibits Robust Antitumor Effects *In Vitro*. A) Cell viability of normal and cancer cells after treated with ConaHA. **B)** Clone formation image of Panc02 and B16F10 cells after treated with ConaHA and its components. **C)** Cell migration-inhibition rate of Panc02 and B16F10 cells after treated with ConaHA and its components at indicated time. **D)** The number of migrated Panc02 and B16F10 cells

were analyzed by Transwell Migration Assay after treated with ConaHA and its components (Scale bar: 50 μm). **E)** Flow cytometry analysis of apoptosis Panc02 or B16F10 cells after different treatment. Data are presented as mean ± SD ($n = 3$). ***$P < 0.001$, **$P < 0.01$, and *$P < 0.05$.

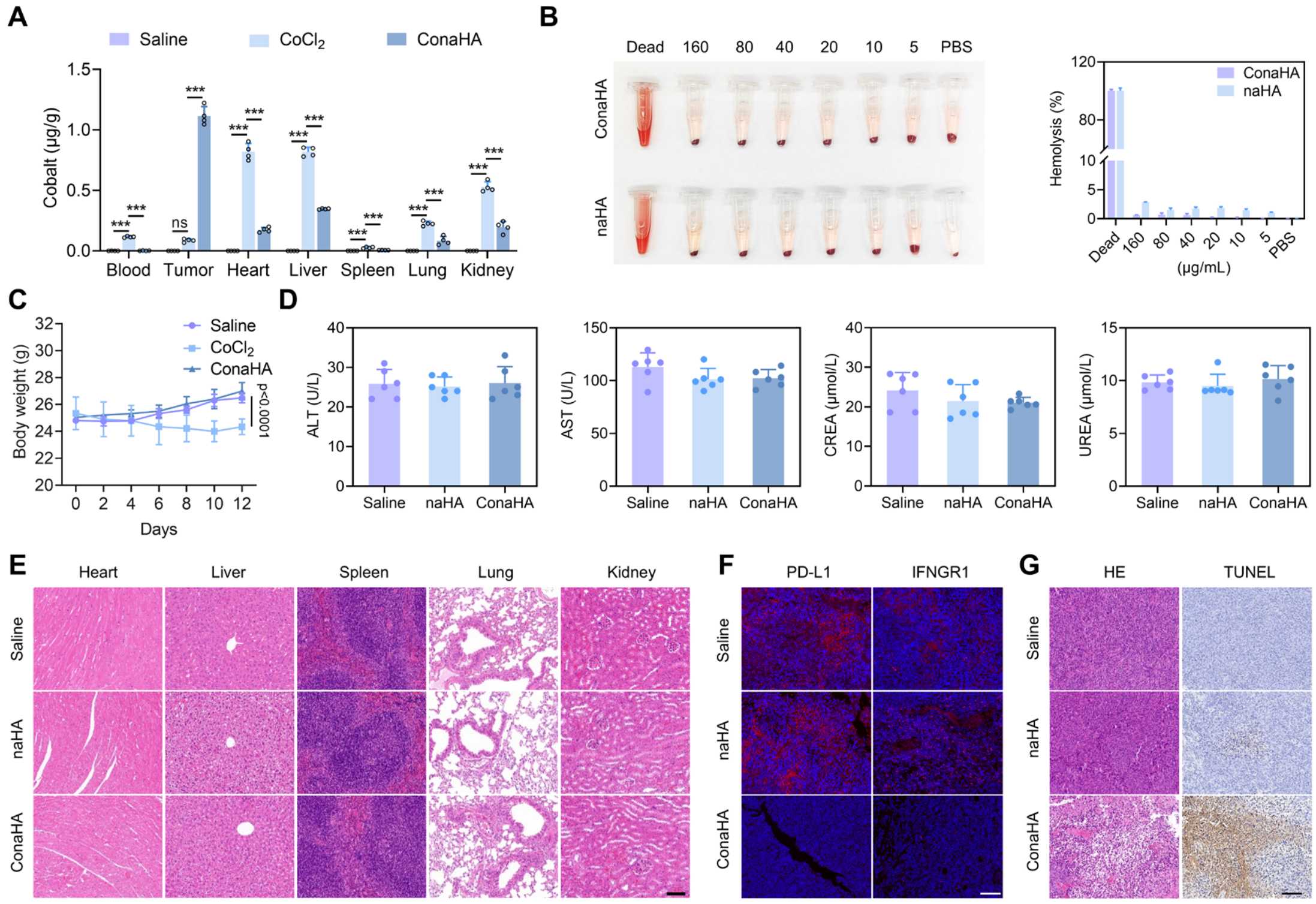

fig. S6. ConaHA Exhibits Good Biocompatibility and Potent Antitumor Efficacy in Panc02 Tumor-Bearing Mice. A) *Ex vivo* cobalt distribution and quantitative analysis of blood, tumor and major organs collected from different groups of mice after administration of Saline, $CoCl_2$ or ConaHA (with the dose based on Cobalt 2.5 mg/kg) intravenously (i.v.) for Panc02-tumor-bearing C57BL/6J mice (n = 4). **B)** Concentration dependent hemolysis and relative digital photo of ConaHA and naHA. H_2O and PBS are set as positive and negative controls, respectively (n = 3). **C)** Saline, $CoCl_2$, ConaHA (with the dose based on Cobalt 2.5 mg/kg) were i.v. administered every other day for C57BL/6J mice (n = 4), and body weight of the mice in the various groups were measured. **D)** The serum ALT, AST, CREA and UREA levels were measured in mice from different treatment groups (n = 6). **E)** H&E staining in each treatment group showed the biocompatibility effect of ConaHA. Scale bar: 100 μm. **F)** Representative immunofluorescence images in tumor tissues of the mice stained with anti-PD-L1 (labeled with Cy3) and anti-IFNGR1 (labeled with Cy3) antibodies in the various groups. Scale bar: 100 μm. **G)** Representative H&E and TUNEL staining images of tumor tissues from different groups. Scale bar: 100 μm. Data are presented as mean ± SD.

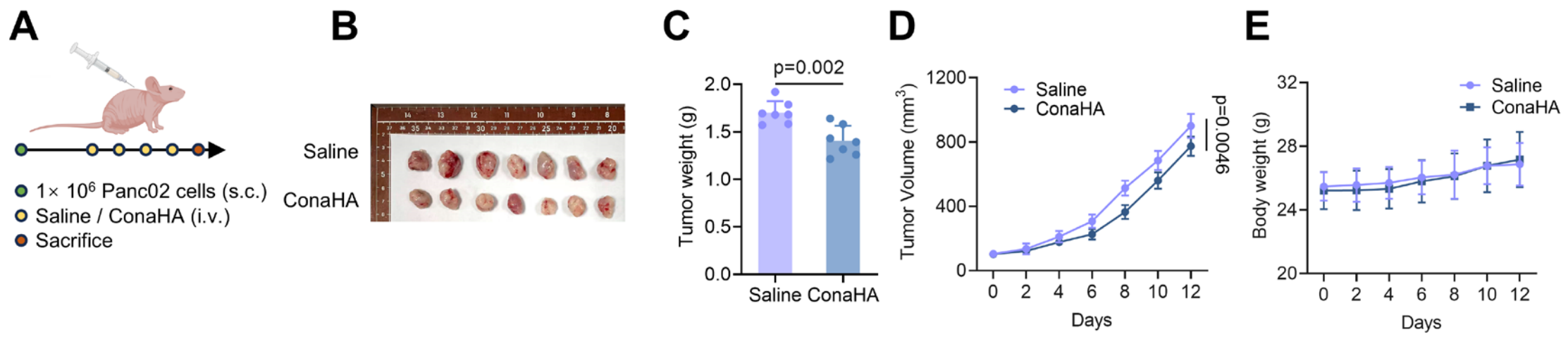

fig. S7. ConaHA Shows Modest Antitumor Activity in Panc02 Tumor-Bearing BALB/c Nude Mice. A) Experimental scheme of the subcutaneous Panc02 tumor models in male BALB/c nude mice. Saline and ConaHA (with the dose based on Cobalt 2.5 mg/kg) were intravenously (i.v.) administered via the tail vein after tumor reached 100 mm^3 (n = 7). **B)** Appearance of tumors in different groups of mice (n = 7). **C)** Average tumor weight of various groups (n = 7). **D)** Mean tumor growth curves of mice (n = 7) subjected to various treatments. **E)** Body weight of the mice in the various groups. Data are presented as mean ± SD (n = 7).

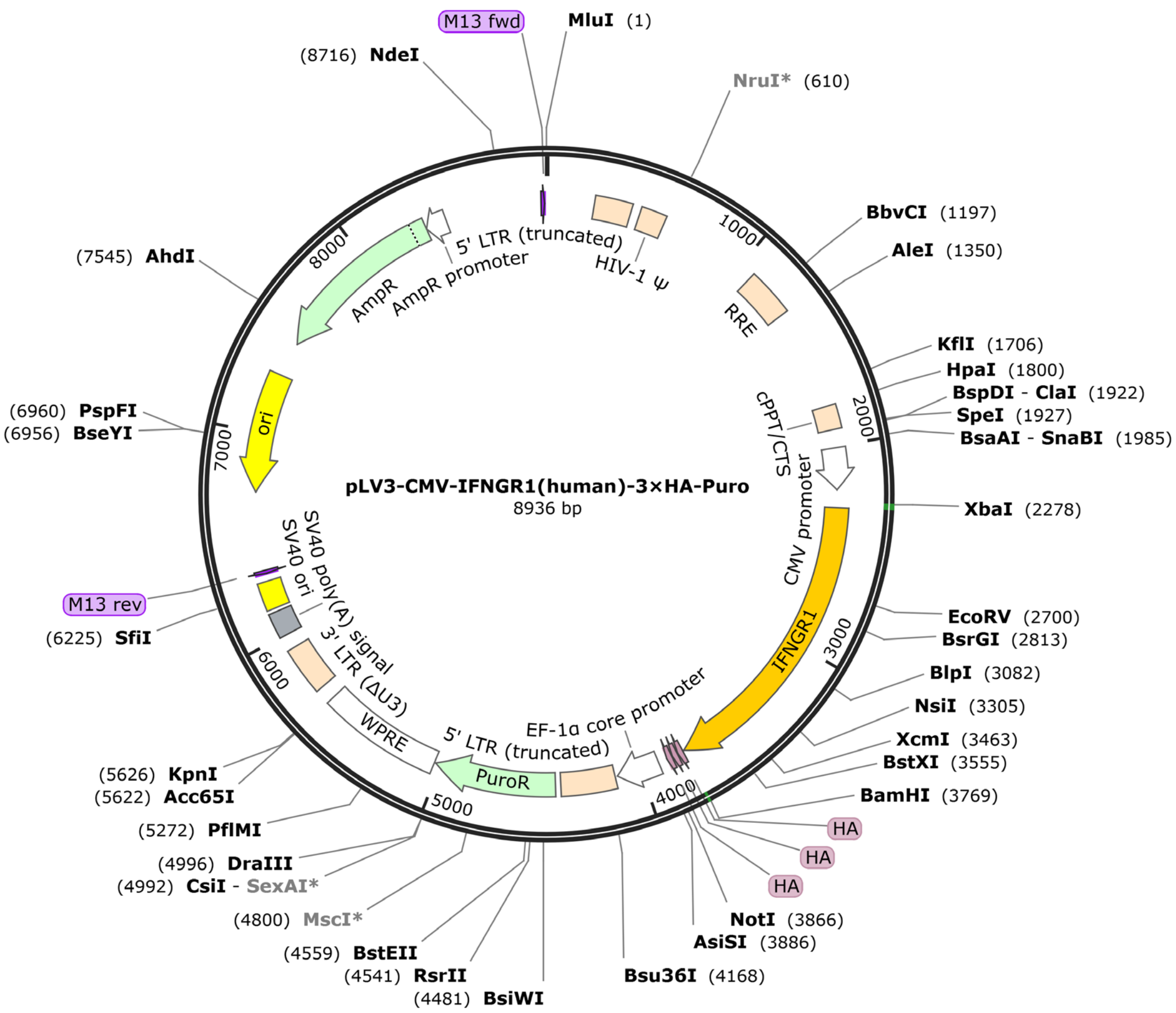

fig. S8. Genetic Map of the Plv3-CMV-IFNGR1-3×HA-Puro Plasmid.

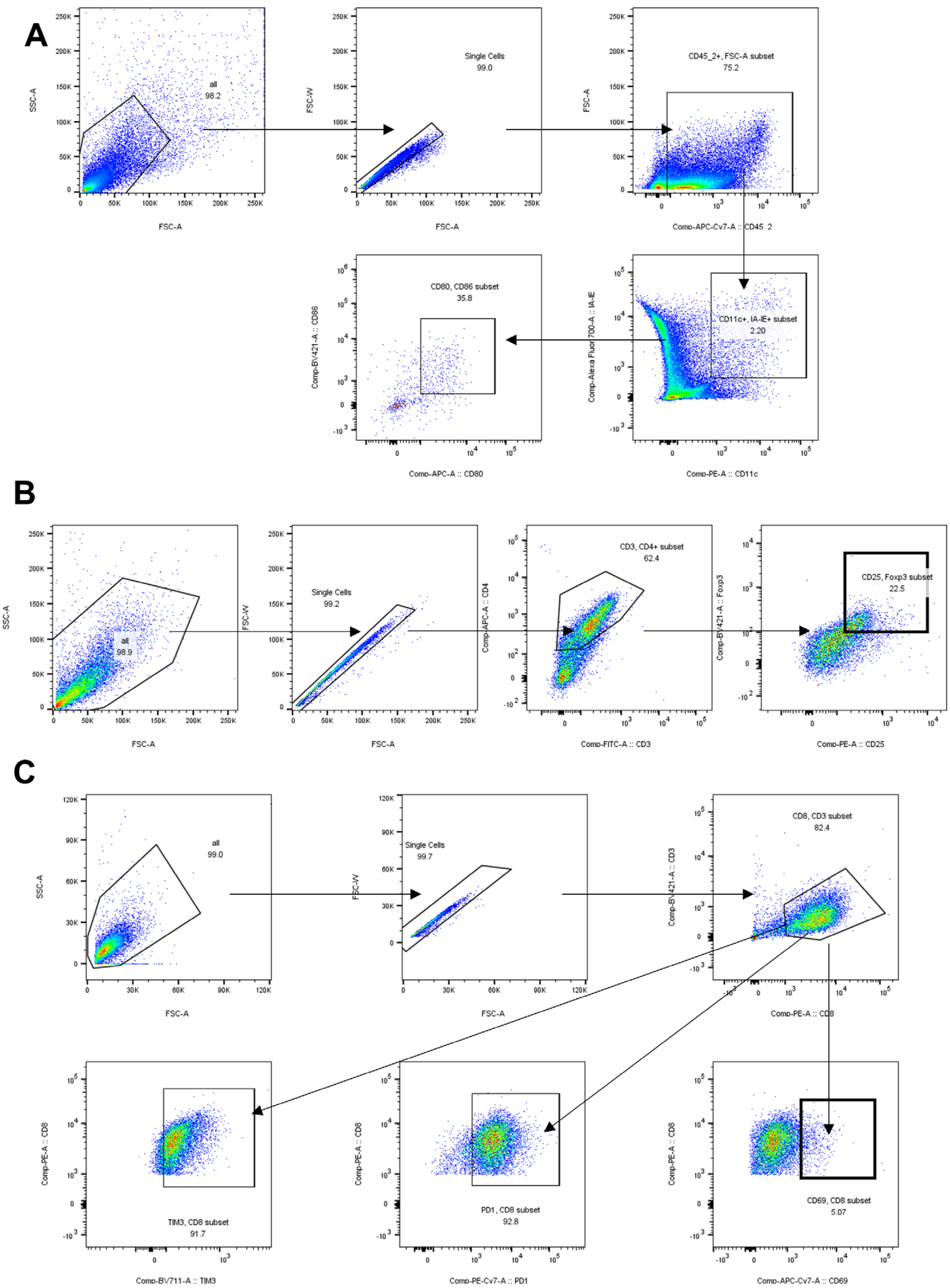
A
all
98.2
SSC-A
FSC-A
Single Cells
99.0
FSC-W
CD45_2+, FSC-A subset
75.2
Comp-APC-Cy7-A :: CD45_2
CD11c+, IA-IE+ subset
2.20
Comp-Alexa Fluor 700-A :: IA-IE
Comp-PE-A :: CD11c
CD80, CD86 subset
35.8
Comp-BV421-A :: CD86
Comp-APC-A :: CD80
B
all
98.9
Single Cells
99.2
CD3, CD4+ subset
62.4
Comp-APC-A :: CD4
Comp-FITC-A :: CD3
CD25, Foxp3 subset
22.5
Comp-BV421-A :: Foxp3
Comp-PE-A :: CD25
C
all
99.0
Single Cells
99.7
CD8, CD3 subset
82.4
Comp-BV421-A :: CD3
Comp-PE-A :: CD8
TIM3, CD8 subset
91.7
Comp-BV711-A :: TIM3
PD1, CD8 subset
92.8
Comp-PE-Cy7-A :: PD1
CD69, CD8 subset
5.07
Comp-APC-Cy7-A :: CD69

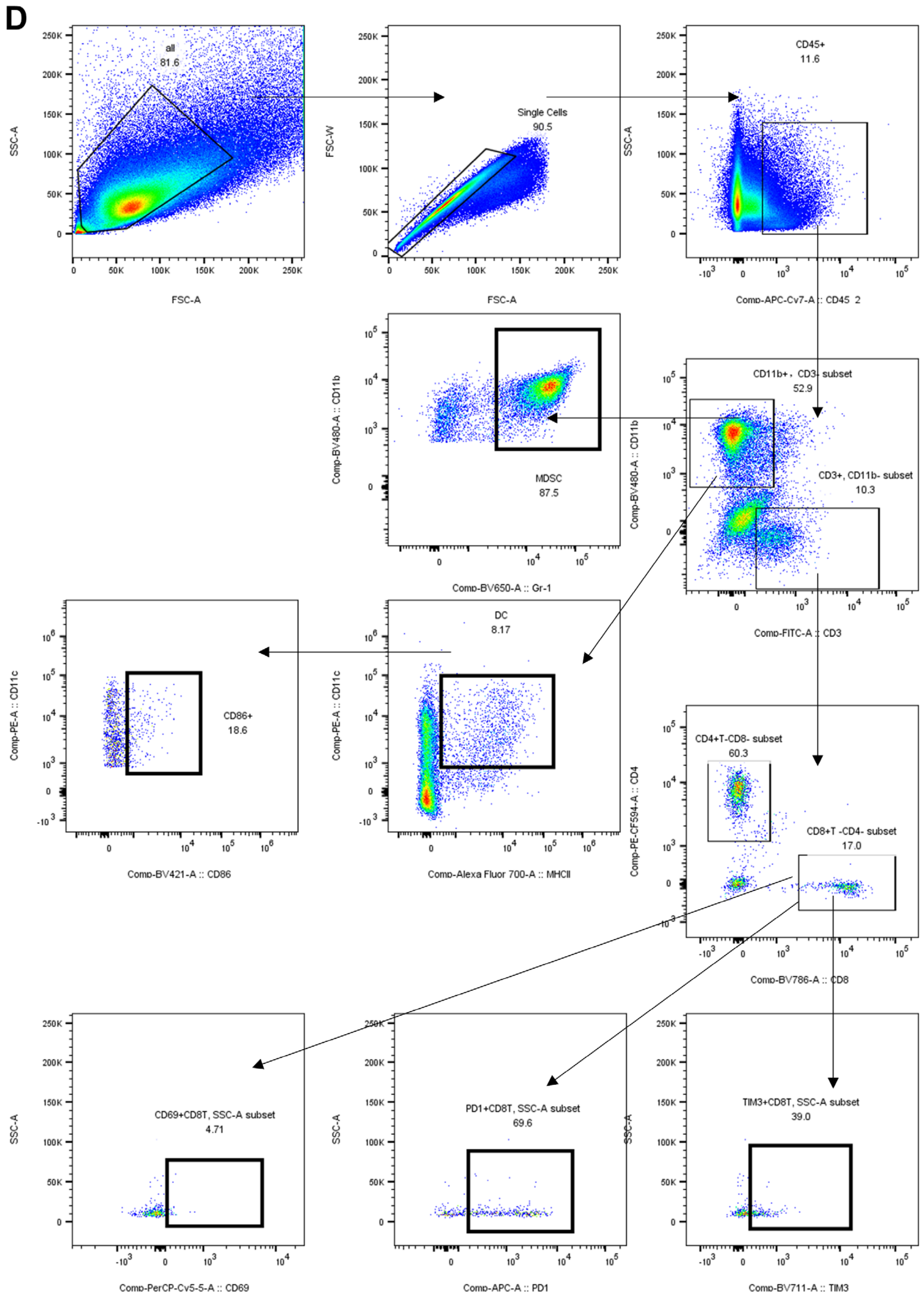
D
all
81.6
SSC-A
FSC-A
Single Cells
90.5
FSC-W
FSC-A
CD45+
11.6
SSC-A
Comp-APC-Cy7-A :: CD45_2
MDSC
87.5
Comp-BV480-A :: CD11b
Comp-BV650-A :: Gr-1
CD11b+，CD3- subset
52.9
CD3+, CD11b- subset
10.3
Comp-BV480-A :: CD11b
Comp-FITC-A :: CD3
CD86+
18.6
Comp-PE-A :: CD11c
Comp-BV421-A :: CD86
DC
8.17
Comp-PE-A :: CD11c
Comp-Alexa Fluor 700-A :: MHCII
CD4+T-CD8- subset
60.3
CD8+T -CD4- subset
17.0
Comp-PE-CF594-A :: CD4
Comp-BV786-A :: CD8
CD69+CD8T, SSC-A subset
4.71
SSC-A
Comp-PerCP-Cy5-5-A :: CD69
PD1+CD8T, SSC-A subset
69.6
SSC-A
Comp-APC-A :: PD1
TIM3+CD8T, SSC-A subset
39.0
SSC-A
Comp-BV711-A :: TIM3

E

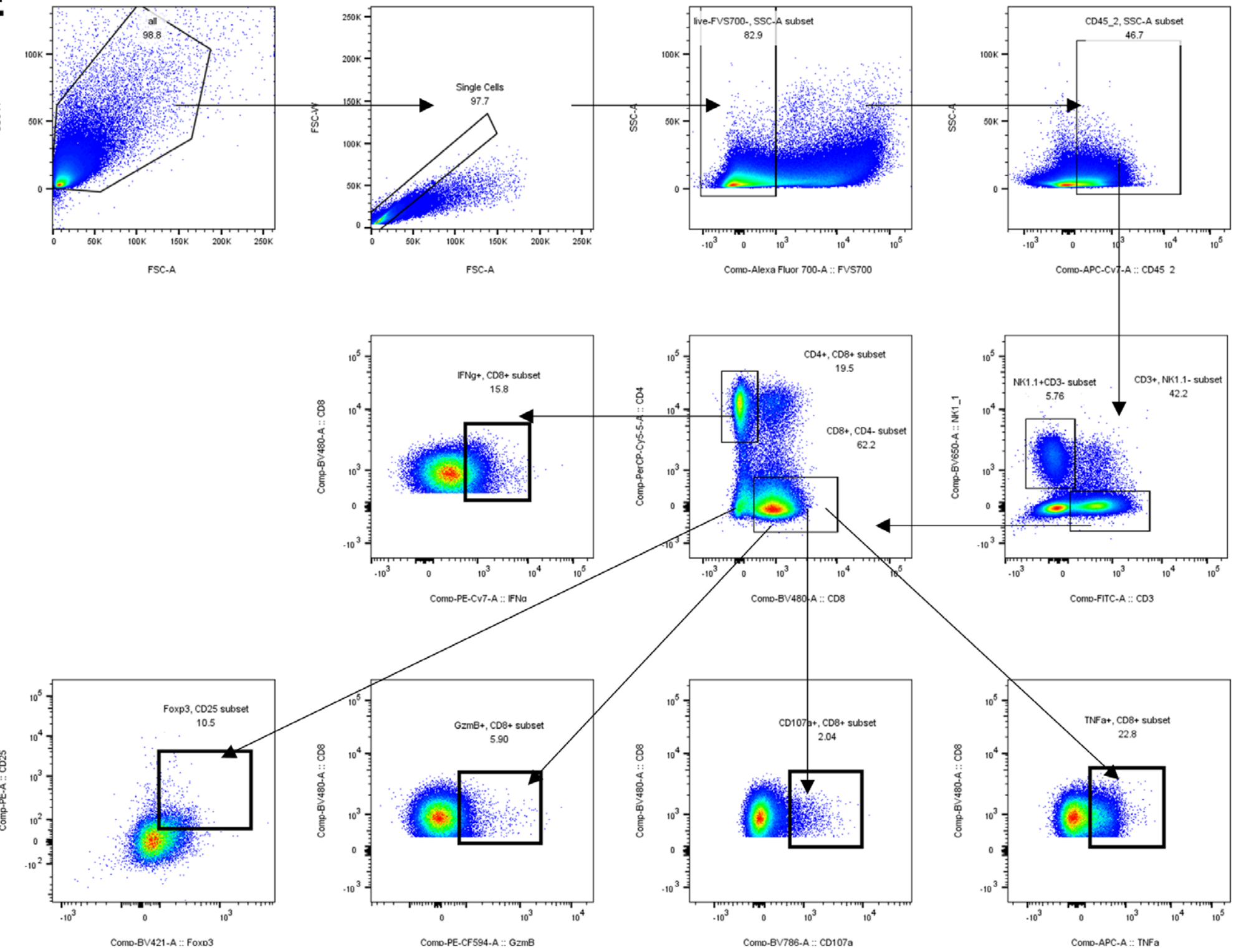

fig. S9. Gating and sorting strategies. A) Representative flow cytometry gating strategies of CD80$^+$CD86$^+$DCs co-cultured with Panc02 cells (Fig. 1G). **B)** Representative flow cytometry gating strategies of CD25$^+$Foxp3$^+$CD4$^+$T cells co-cultured with Panc02 cells (Fig. 1H). **C)** Representative flow cytometry gating strategies of PD-1$^+$CD8$^+$ T cells, TIM-3$^+$CD8$^+$ T cells and CD69$^+$CD8$^+$ T cells co-cultured with Panc02 cells (Fig. 2E, F, I). **D, E)** Representative flow cytometry gating and sorting strategies of tumor-infiltrating immune cells in Panc02, MC38 and B16F10 mouse models (Fig. 6, Fig. 7F, K).

Table S1 Reagents for experiments

Reagents	Clone	Source	Identifier
APC/Cyanine7 anti-mouse CD45.2	104	Biolegend	109824
FITC anti-mouse CD3	17A2	Biolegend	100204
PE/Dazzle™ 594 anti-mouse CD4	RM4-5	Biolegend	100566
Brilliant Violet 785 anti-mouse CD8a	53-6.7	Biolegend	100750
PerCP/Cyanine5.5 anti-mouse CD69	H1.2F3	Biolegend	104522
APC anti-mouse PD-1	29F.1A12	Biolegend	135210
Brilliant Violet 711™ anti-mouse TIM-3	RMT3-23	Biolegend	119727
Brilliant Violet 510™ anti-mouse CD11b	M1/70	Biolegend	101263
PE anti-mouse CD11c	N418	Biolegend	117308
Brilliant Violet 650™ anti-mouse Gr-1	RB6-8C5	Biolegend	108442
Alexa Fluor® 700™ anti-mouse I-A/I-E	M5/114.15.2	Biolegend	107622
PE/Cyanine7 anti-mouse NK-1.1	PK136	Biolegend	108714
BD Horizon™ RB705 Rat Anti-Mouse CD4	GK1.5	BD Pharmingen	570257
Brilliant Violet 510™ anti-mouse CD8	53-6.7	Biolegend	100752
BD Horizon™ BV786 Rat Anti-Mouse CD107a	1D4B	BD Pharmingen	564349
PE anti-mouse CD25	3C7	Biolegend	101904
Brilliant Violet 650™ anti-mouse NK-1.1	PK136	Biolegend	108736
PE/Cyanine7 anti-mouse IFN-γ	XMG1.2	Biolegend	505826
PE/Dazzle™ 594 anti-human/mouse Granzyme B	QA16A02	Biolegend	372216
Brilliant Violet 421™ anti-mouse Foxp3	MF-14	Biolegend	126419
APC anti-mouse TNFα	TN3-19.12	Biolegend	506108
APC anti-mouse CD4	RM4-5	Biolegend	100516
PE Mouse Anti-Ki-67	B56	BD Pharmingen	567719
Brilliant Violet 421™ anti-mouse CD86	GL-1	Biolegend	105123
APC anti-mouse CD80	16-10A1	Biolegend	104713
Purified anti-mouse CD16/32	93	Biolegend	101302

Table S2 Oligonucleotide primers used for qPCR

Name	Species	Primer Sequences
IDO1-F	Mus musculus	GCAGACTGTGTCCTGGCAAACT
IDO1-R	Mus musculus	AGAGACGAGGAAGAAGCCCTTG
IFNGR1-F	Mus musculus	TGACGGGAGCACCTGTTACAC
IFNGR1-R	Mus musculus	TTTCGACCGTATGTTTCGTATGTAG
GAPDH-F	Mus musculus	CATCACTGCCACCCAGAAGACTG
GAPDH-R	Mus musculus	ATGCCAGTGAGCTTCCCGTTCAG

Table S3 Plasmid used for transfection

Plasmid name	Backbone	Insert (species)	Fusion tag	Mutation
pLV3-CMV-IFNGR1-3×HA-Puro	pLV3-CMV-Puro	IFNGR1 (Human)	3×HA	WT
pLV3-CMV-IFNGR1(H83A)-3×HA-Puro	pLV3-CMV-Puro	IFNGR1 (Human)	3×HA	H83A
pLV3-CMV-IFNGR1(H461A)-3×HA-Puro	pLV3-CMV-Puro	IFNGR1 (Human)	3×HA	H461A

Table S4 Oligonucleotides used for point mutation

Name	Species	Sequence (5'→3')
IFNGR1_H83A_F	Human	tggattgatgcctgcatcaatatttctcatGCttattgtaatatttctgatcatgttggtgatccatcaaattctc
IFNGR1_H83A_R	Human	gatgcaggcatcaatccattctg
IFNGR1_H461A_F	Human	aaccaGCtgtgctagtggatctacttgtggatgatagc
IFNGR1_H461A_R	Human	atccactagcacaGCtggtttatcataaccaaaggaggtgggg
